# Aluminum-activated malate transporter family member CsALMT6 mediates fluoride resistance in tea plants (*Camellia sinensis*)

**DOI:** 10.1093/hr/uhae353

**Published:** 2024-12-12

**Authors:** Qinghui Li, Ruiming Zhang, Xinlong Hu, Dejiang Ni, Yuqiong Chen, Mingle Wang

**Affiliations:** National Key Laboratory for Germplasm Innovation and Utilization of Horticultural Crops, College of Horticulture and Forestry Sciences, Huazhong Agricultural University, Wuhan 430070, China; National Key Laboratory for Germplasm Innovation and Utilization of Horticultural Crops, College of Horticulture and Forestry Sciences, Huazhong Agricultural University, Wuhan 430070, China; National Key Laboratory for Germplasm Innovation and Utilization of Horticultural Crops, College of Horticulture and Forestry Sciences, Huazhong Agricultural University, Wuhan 430070, China; National Key Laboratory for Germplasm Innovation and Utilization of Horticultural Crops, College of Horticulture and Forestry Sciences, Huazhong Agricultural University, Wuhan 430070, China; National Key Laboratory for Germplasm Innovation and Utilization of Horticultural Crops, College of Horticulture and Forestry Sciences, Huazhong Agricultural University, Wuhan 430070, China; National Key Laboratory for Germplasm Innovation and Utilization of Horticultural Crops, College of Horticulture and Forestry Sciences, Huazhong Agricultural University, Wuhan 430070, China

## Abstract

Tea plant is a fluoride (F)-hyperaccumulator, which poses a potential threat to human health *via* tea consumption. Reducing F accumulation in fresh tea leaves is crucial for enhancing the safety of tea production at its source. This study aims to isolate novel genes responsible for F accumulation or transport in tea plants. We identified an aluminum (Al)-activated malate transporter gene, *CsALMT6*, which was hypothesized to be a candidate for differential F accumulation in *Camellia sinensis*, by employing a combination of transcriptome-wide association study and genome-wide identification of the *CsALMT* gene family. *CsALMT6* exhibited high expression levels in old leaves, and its expression was significantly upregulated in tea plants subjected to F-stress conditions. Furthermore, heterologous expression of *CsALMT6* in yeast, *Arabidopsis*, and *Populus* conferred F tolerance. However, the expression of F-tolerant hub genes, *CsFEX1* and *CsFEX2*, remained unaffected in *CsALMT6*-silenced tea plants. Additionally, under F toxicity conditions, the transcription of *CsALMT6* was negatively associated with F accumulation in tea plants. In conclusion, *CsALMT6* plays a vital role in reducing F accumulation in *C. sinensis*, thus conferring F tolerance to plant cells.

## Introduction

Fluorine, a widely occurring element in the environment, is predominantly present as a fluoride (F) compound. Moderate F intake is beneficial for human health, particularly in promoting the formation of bones and teeth [1]. However, excessive F consumption leads to various forms of fluorosis, including dental fluorosis, skeletal fluorosis, and other symptoms [2]. In plants, F toxicity induces chlorosis and marginal necrosis, such as tipburn, scorching, or lesions in leaves [3]. Furthermore, F stress negatively affects seed germination, photosynthesis, nutrient uptake, and protein accumulation while also inhibiting reproductive characteristics, resulting in growth retardation [4]. Compared with *Zea mays* and *Glycine max*, *Sorghum vulgare* exhibits greater resistance to F stress [5]. To maintain F concentration at the desired physiological level, plants have evolved sophisticated and precise mechanisms for F uptake, accumulation, tolerance responses, and detoxification [4]. Fluoride exporter (FEX) has been confirmed as the primary F defense mechanism in *Arabidopsis thaliana*, mainly involved in the efflux of toxic F ions from cells through its membrane transport activity [6, 7]. Additionally, chloride channel 2 (*OsCLC2*), rather than *OsCLC1*, was responsible for F export in F-tolerant *Oryza sativa* cultivars [8]. Apart from this, until recently, little was known about whether plants detoxify F through other mechanisms. Therefore, further exploration of novel genes and new mechanisms involved in plant F tolerance is necessary.

Aluminum (Al)-activated malate transporters (ALMTs) are involved in numerous physiological processes in flora, including metal tolerance [9], stomatal movement [10], anion homeostasis [11], seed development, and fruit quality [12]. *TaALMT1* was the first *ALMT* gene isolated from the root tips of *Triticum aestivum* [13], which can be induced by Al^3+^ stress, release malate anion, and confer tolerance to Al^3+^ toxicity in wheat [14, 15]. Furthermore, *TaALMT1* is permeable not only to malate but also to Cl^−^, SO_4_^2−^, and NO_3_^−^ in the absence of extracellular Al^3+^ [16]. *AtALMT9* functions as a malate-activated Cl^−^ channel involved in regulating stomatal movement in *A. thaliana* [17]. *AtALMT4*, *AtALMT6*, *AtALMT12*, and *HvALMT1* are also involved in guard cell regulation [11, 18]. *MdALMT9* and *SlALMT9* are responsible for malic acid accumulation in apple and tomato, respectively [19, 20]. Moreover, *AcALMT1* and *PpALMT9* are involved in the transport and accumulation of citrate in kiwifruit and peach fruits, respectively [21, 22]. Additionally, *CsALMT5* was hypothesized to be induced by Al^3+^ toxicity within the pollen tube in tea plants [23]. However, it remains unclear whether ALMTs are involved in F tolerance in plants.

*Camellia sinensis* is an F hyper-accumulator, with high F concentrations typically found in the leaves, particularly in mature and old leaves [24]. Several factors influence F accumulation, including soil F content, tea plant cultivars, leaf maturity, and cultivation practices [25, 26]. Previous research has demonstrated that F toxicity negatively impacts the synthesis of key compounds in tea leaves, such as polyphenols, amino acids, and aromatic substances, leading to a reduction in tea quality [27]. Tea plants possess specific F tolerance mechanisms that facilitate F accumulation and detoxification [28]. A recent study revealed that *CsABCB9* plays a role in transporting F ions from the chloroplast to the cytosol, thereby mitigating F toxicity in the chloroplast [29]. Additionally, CsFEXs, which are localized in the plasma membrane, have been implicated in the efflux of F ions, reducing F accumulation and alleviating F toxicity in *C. sinensis* [30, 31]. Furthermore, F toxicity induces differential expression of genes related to plant hormone metabolism and signaling pathways [32]. Despite these findings, the specific mechanisms underlying F accumulation, detoxification, and toxicity remain to be fully elucidated. Therefore, further research is necessary to identify the key genes responsible for F transport, tolerance, and accumulation, and to elucidate their specific biological functions.

**Figure 1:**
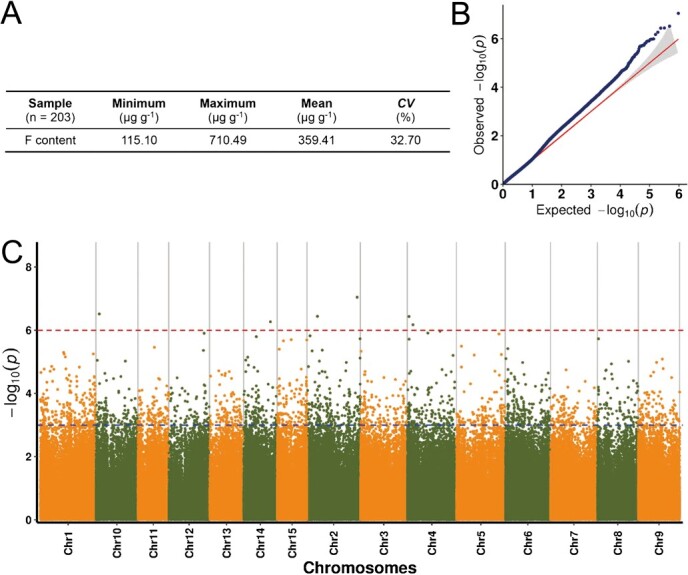
TWAS for F content in *C. sinensis*. (A) Variation of F content in *C. sinensis* leaves. (B) Quantile-quantile (QQ) plot of the TWAS analysis using the F content as phenotypic data. (C) Manhattan plot of the TWAS using the F content as phenotypic data. The blue- and red-dashed lines in the Manhattan plot indicate the significance thresholds (−log_10_*P* = 3.00 and 6.00), respectively.

In this study, a transcriptome-wide association study (TWAS) was employed to explore novel genes involved in F differential accumulation, utilizing transcriptome data and F content from 203 *C. sinensis* germplasm resources. Three candidate genes, *CsALMT1*, *CsALMT2*, and *CsALMT6*, were screened, followed by a genome-wide identification of *CsALMT* family members and an investigation of their expression profiles in different tissues or in response to F toxicity. Based on the expression profiles of *CsALMT*s, *CsALMT6* was further speculated to be a candidate gene for F tolerance in *C. sinensis*. To confirm this hypothesis, the biological functions of *CsALMT6* involved in F tolerance were comprehensively addressed using transgenic yeast, *Arabidopsis*, and *Populus* systems, and the F uptake, tolerance, and accumulation of the transgenic plants were analyzed. Furthermore, virus-induced gene silencing (VIGS) of *CsALMT6* and RNA-sequencing were performed to analyze the global transcriptome change in response to F toxicity in tea plants. Additionally, the relationship between *CsALMT6* expression and F accumulation in low-F and high-F tea plant cultivars was investigated. These findings contribute to the elucidation of the molecular mechanism of *CsALMT6*-mediated F resistance in *C. sinensis* and may facilitate future molecular breeding efforts aimed at developing low-F tea plant cultivars and improving the safety of tea beverages.

## Results

### TWAS on the F content in tea plants

A total of 203 *C. sinensis* germplasm resources were employed to determine the F content in tea leaves, which exhibited substantial variation, ranging from 115.10 to 710.49 μg·g^−1^ (Fig. 1A). To identify the key genes associated with F content, we performed a TWAS, and the generalized linear model (GLM) provided a good approximation of the expected cumulative distribution of *P* values (Fig. 1B and C). Further analysis indicated that three *CsALMT* genes may be promising candidates based on the TWAS analysis (Table 1). According to their homology to *A. thaliana* ALMT family members, we designated CSS0030958, CSS0015822, and CSS0039766 as *CsALMT1*, *CsALMT2*, and *CsALMT6*, respectively (Supplementary Table S1).

**Table 1:** Annotations of the three candidate *CsALMT* genes

**Gene ID**	**log10P**	**Chromosomal position**	**Start**	**End**	**Gene annotation**
CSS0030958	3.53	Chr11	115 378 309	115 382 681	*CsALMT1*
CSS0015822	3.53	Chr11	115 281 306	115 286 528	*CsALMT2*
CSS0039766	3.49	Chr14	61 165 154	61 173 072	*CsALMT6*

### Identification of *CsALMT* family genes in *C. sinensis*

To investigate other potential members of *CsALMT*s involved in F accumulation, a total of 16 *CsALMT*s were identified from the *C. sinensis* cv. 'Shuchazao' genome. These genes were designated *CsALMT1* to *CsALMT16* based on their homology with *AtALMT*s (Supplementary Table S1). Analysis of the physicochemical properties of *CsALMTs* revealed that their lengths ranged from 408 (*CsALMT13*) to 793 amino acids (*CsALMT4*). The molecular weights (MWs) varied from 45.85 (*CsALMT13*) to 88.72 (*CsALMT4*) kDa, and the predicted isoelectric point (pI) values ranged from 5.66 (*CsALMT5*) to 8.98 (*CsALMT14*). Notably, *CsALMT14* had a pI value of 8.98, indicating that this protein was more basic than the other 15 members. The total average hydrophobicity ranged from −0.152 (*CsALMT9*) to 0.215 (*CsALMT16*). The grand average of hydropathicity (GRAVY) values for *CsALMT4*, *CsALMT5*, *CsALMT9*, and *CsALMT15* were less than zero, suggesting their hydrophilic nature. Furthermore, subcellular localization prediction indicated that all *CsALMT* members were localized in the plasma membrane (Supplementary Table S1).

### Phylogenetic, chromosomal location, and conserved motifs analyses

**Figure 2:**
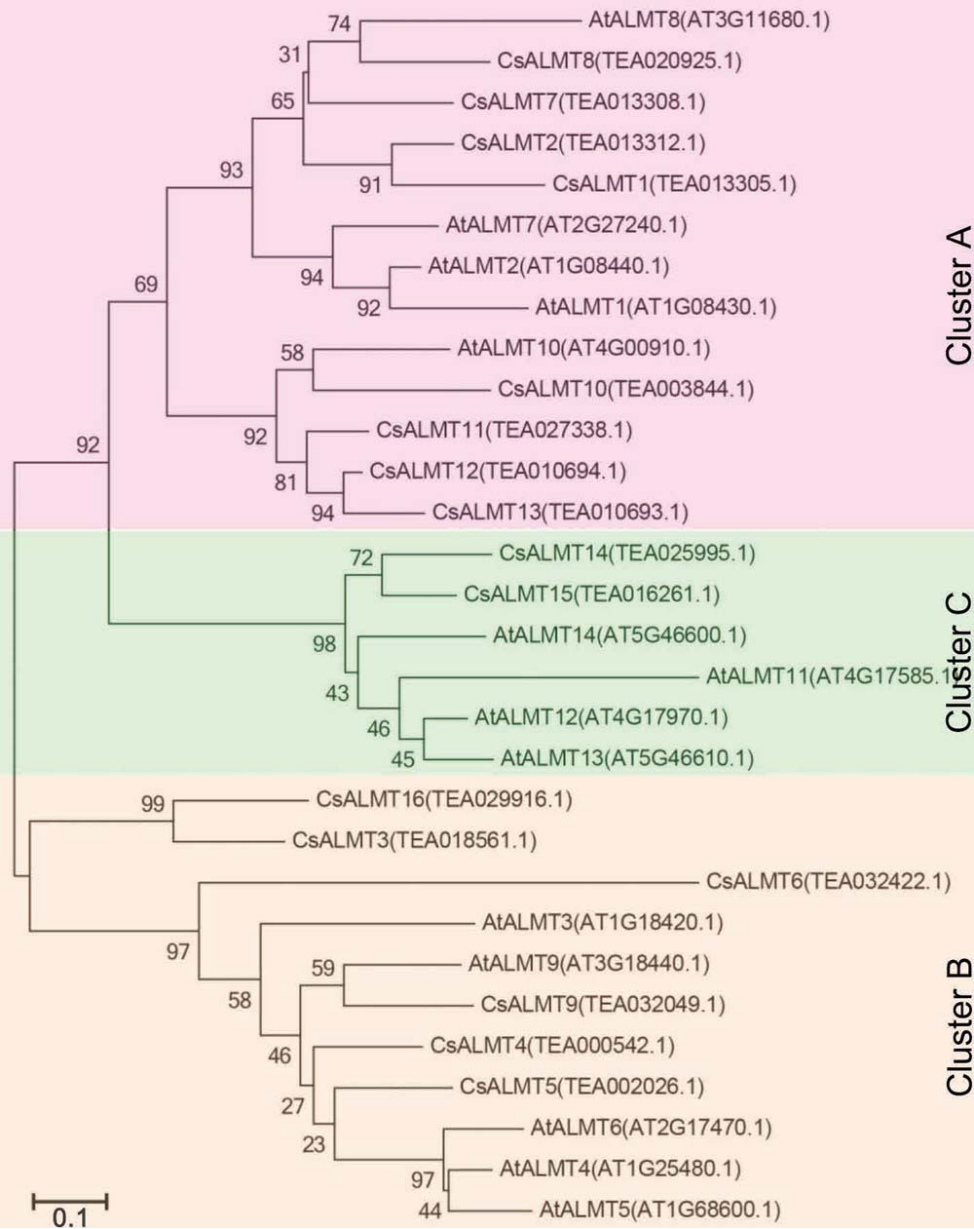
Phylogenetic trees of ALMTs between *C. sinensis* and *A. thaliana*.

To elucidate the evolutionary relationships among ALMTs, a phylogenetic tree was constructed utilizing the 16 CsALMTs and 14 *A. thaliana* ALMTs. The analysis revealed that all CsALMTs were classified into three primary clusters: A, B, and C (Fig. 2). Significantly, CsALMT1 and CsALMT2 were categorized within the largest subfamily A, which encompassed eight CsALMTs. Conversely, CsALMT6 diverged into a distinct branch within subfamily B, indicating potential functional differentiation between *CsALMT6* and other members of the ALMT family.

Chromosomal location analysis indicated that the 16 *CsALMT* genes were distributed on 8 chromosomes, exhibiting an uneven distribution (Supplementary Fig. S1). Specifically, four genes were located on both chromosomes 11 and 14, two genes on chromosome 13, and one gene each on chromosomes 1, 3, 10, 12, and 15. Furthermore, *CsALMT10* was not found on the chromosomes but was anchored on contig331. Although the 16 *CsALMT*s were distributed across the chromosomes, several genes clustered closely together. For instance, *CsALMT1*, *CsALMT2*, and *CsALMT7*, which were closer to each other in the phylogenetic tree, were located on chromosome 11.

Analysis of the *CsALMT*s revealed highly conserved motifs (Supplementary Fig. S2A), with 10 motifs identified, of which motifs 1, 2, 3, 7, 8, and 9 exhibited the highest level of conservation within the *CsALMT* family (Supplementary Fig. S3). Furthermore, the *CsALMT* genes displayed a generally consistent structure, with a significant proportion containing six exons (Supplementary Fig. S2B), suggesting a substantial degree of conservation throughout the evolutionary process.

### Expression profiles of *CsALMT*s in different tissues of *C. sinensis*

**Figure 3:**
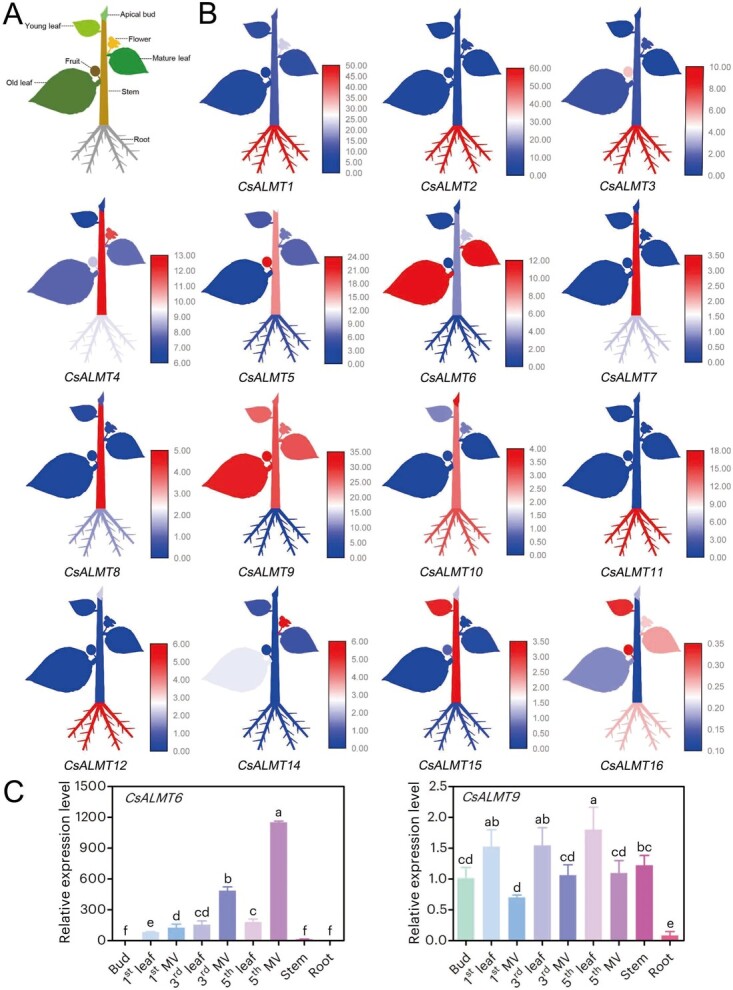
Expression profiles of *CsALMT*s in various tissues of *C. sinensis*. (A) Schematic diagram depicting different tissues in *C. sinensis*. (B) Expression levels of the 16 *CsALMT* family members across eight tissues of *C. sinensis*. Red indicates high gene transcription levels, while blue represents low gene transcription levels. (C) Relative expression levels of *CsALMT6* and *CsALMT9* in different tissues. The expression levels of *CsALMT6* and *CsALMT9* in the bud were normalized to 1.0, respectively. MV: major vein. Data are expressed as means ± standard deviation (SD) (*n* = 3), and different letters indicate significant differences at *P* < 0.05.

To elucidate the potential biological functions of *CsALMT*s in *C. sinensis*, we initially analyzed the expression patterns of 16 *CsALMT*s across eight tissues (Supplementary Table S2). The *CsALMT*s displayed differential expression among the examined tissues (Fig. 3A and B). Notably, high transcript levels were observed for *CsALMT1*, *2*, *3*, *11*, and *12* in the root, while *CsALMT4*, *7*, *8*, and *15* exhibited elevated expression in the stem. Furthermore, *CsALMT13* was undetectable in all tissues, suggesting that its expression may be restricted to specific developmental stages. Additionally, it is noteworthy that *CsALMT6* and *CsALMT9* were highly expressed in mature and old leaves. Previous research has demonstrated that F primarily accumulates in the old leaves of tea plants. Consequently, we hypothesize that *CsALMT6* and *CsALMT9* may contribute to F accumulation in *C. sinensis*.

To more precisely evaluate the expression patterns of *CsALMT6* and *CsALMT9* in different tissues, quantitative real-time polymerase chain reaction (qRT-PCR) was conducted on samples from various leaf positions [i.e. bud, first leaf, first major vein (MV), third leaf, third MV, fifth leaf, fifth MV], stem, and root (Fig. 3C). The expression level of *CsALMT6* exhibited significant differences across these tissues (*P* < 0.05), with high levels maintained in the first MV, third MV, and fifth MV, suggesting the potential involvement of *CsALMT6* in F transport. Furthermore, *CsALMT9* displayed relatively high expression levels in the first leaf, third leaf, and fifth leaf, indicating its possible participation in F metabolism.

### F content in different leaf positions of two *C. sinensis* cultivars

**Figure 4:**
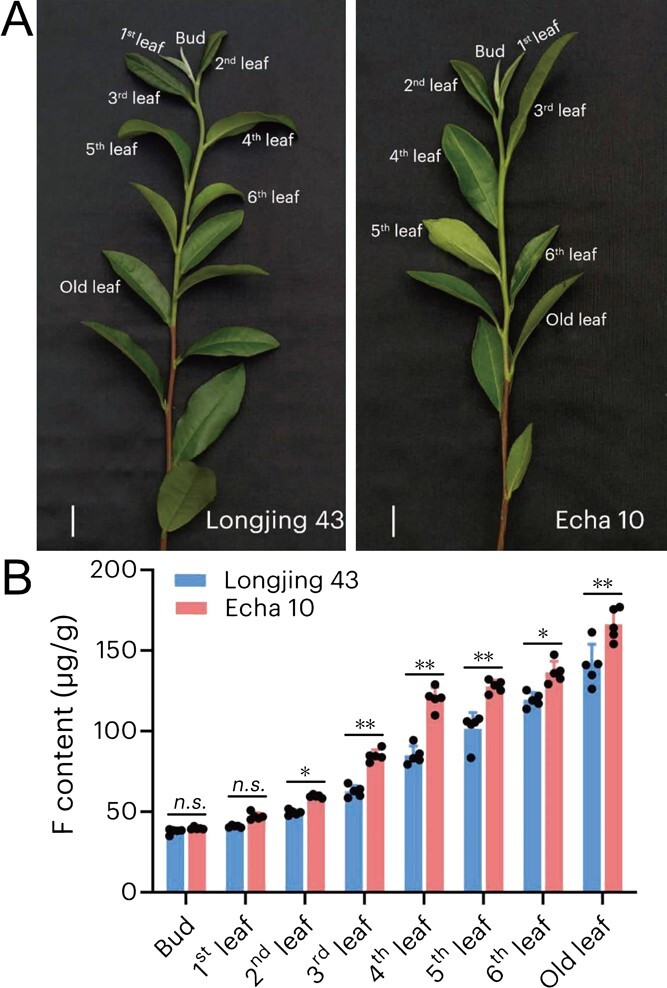
F content in different leaf positions of tea plants. (A) Schematic diagram of the sampling sites in tea plants. Bar = 2 cm. (B) F content in different leaf positions between 'Longjing 43′ and 'Echa 10′. Data are expressed as means ± standard deviation (SD) (*n* = 5), and asterisks indicate significant differences between the two cultivars at *P* < 0.05 (*) or *P* < 0.01 (**). *n.s.*: no significant difference.

To further validate our hypothesis regarding the role of *CsALMT6* in F transport, two tea plant cultivars ('Longjing 43′ and 'Echa 10′) were selected, and F contents were measured in their leaves at varying maturity levels (Fig. 4A). The F contents exhibited a gradual increase with leaf development in both *C. sinensis* cultivars (Fig. 4B). Moreover, the F content was lowest in the bud and highest in the old leaves. Notably, the F content in 'Echa 10′ was significantly higher than that in 'Longjing 43′ from the second leaf to the old leaf (*P* < 0.05 or *P* < 0.01). Consequently, we selected one bud with two leaves (i.e. shoot) as sampling sites for conducting the subsequent qRT-PCR assay under high concentrations of NaF treatments.

### Expression patterns of *CsALMT* family genes in response to F treatments in *C. sinensis*

**Figure 5:**
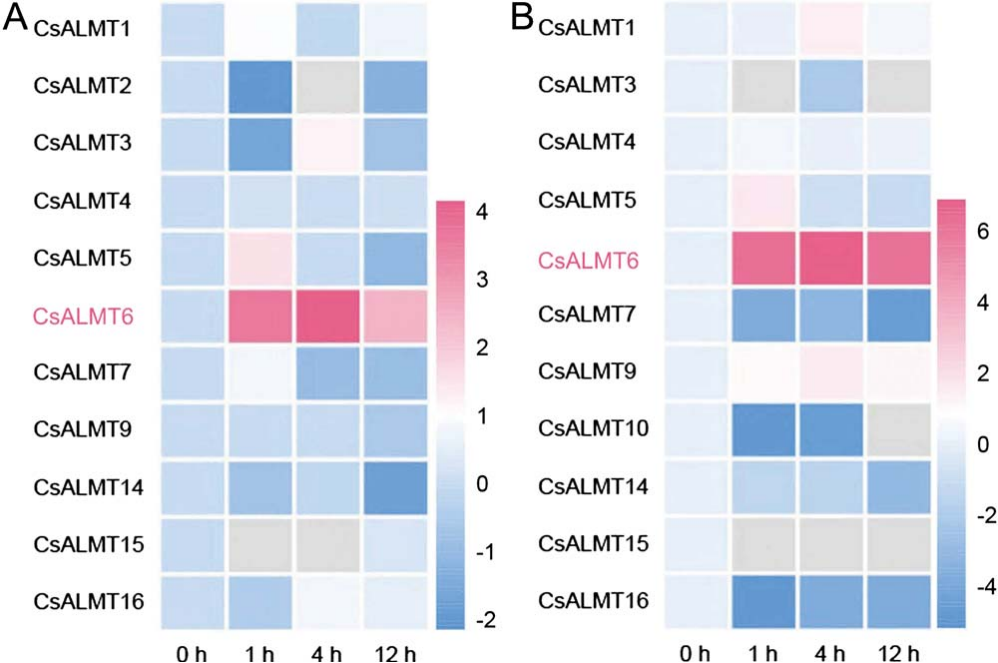
Expression patterns of *CsALMT* family genes in *C. sinensis* shoots under F treatments. The heatmaps depict *CsALMT*s expression levels in (A) 'Longjing 43′ and (B) 'Echa 10′. The color bar represents the log2-transformed 2^-ΔΔCT^ values, while gray indicates no detectable expression.

To comprehensively investigate the responses of *CsALMT*s to F stress, a qRT-PCR assay was performed to detect the expression of *CsALMT*s in the shoots of 'Longjing 43′ and 'Echa 10′ exposed to high concentrations of NaF treatments. *CsALMT* family members exhibited different expression patterns in the shoots of the two cultivars when exposed to F treatments (Fig. 5 and Supplementary Table S3). The expression levels of *CsALMT5* increased by 2.93- and 3.28-fold in 'Longjing 43′ and 'Echa 10′ after 1 h of F treatments, respectively; the expression levels of *CsALMT3* in 'Longjing 43′, and *CsALMT1* and *CsALMT9* in 'Echa 10′ also showed a slight increase. *CsALMT2* was only detectable in 'Longjing 43′, while *CsALMT10* was only detectable in 'Echa 10′. Conversely, *CsALMT11*, *12* and *13* were undetectable in both *C. sinensis* cultivars. Notably, the expression levels of *CsALMT6* were significantly upregulated at 4 h under F treatments in both 'Longjing 43′ and 'Echa 10′ (*P* < 0.05), indicating that *CsALMT6* may be involved in the response to F tolerance in *C. sinensis*.

### Heterologous expression of *CsALMT6* confers tolerance to F toxicity in transgenic yeast

**Figure 6:**
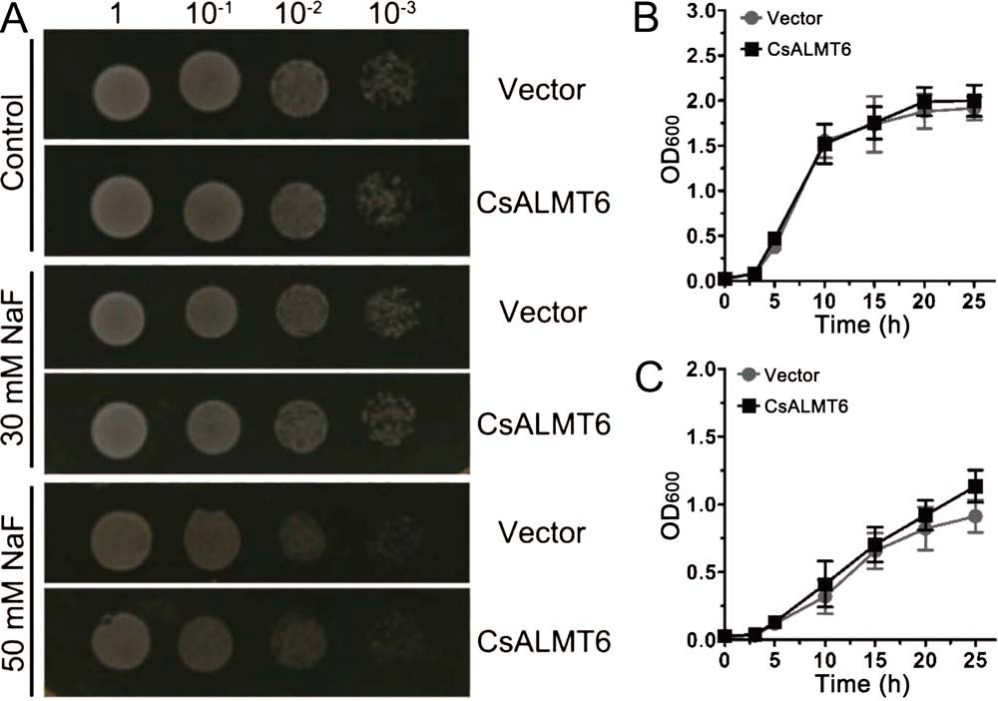
F tolerance of *CsALMT6* transgenic yeast. (A) Phenotypes of yeast cells transformed with pYES2-*CsALMT6* (i.e. CsALMT6) or empty vector (i.e. vector) under different concentrations of NaF treatments [i.e. 0 (Control), 30 and 50 mM] for two days. Growth curves of yeast cells harboring pYES2-*CsALMT6* or empty vector under (B) 0 mM and (C) 50 mM NaF treatments. Vector and CsALMT6 refer to the transgenic yeast harboring pYES2 and pYES2-*CsALMT6*, respectively. Data are presented as means ± SD (*n* = 4). The asterisk indicates a significant difference between the yeast cells harboring pYES2-*CsALMT6* and the empty vector, respectively (*P* < 0.05).

To preliminarily clarify the function of *CsALMT6* in F tolerance, a yeast expression vector (i.e. pYES2-*CsALMT6*) was constructed and transformed into the *Saccharomyces cerevisiae* INVSc1 strain. When grown on YPDA medium plates without NaF addition (i.e. Control), both vector and CsALMT6 yeast cells exhibited similar growth rates (Fig. 6A and B). Under NaF treatments (i.e. 30 and 50 mM), the growth rates of vector and CsALMT6 were inhibited; however, the *CsALMT6*-overexpressing cells demonstrated a higher growth rate than the vector when exposed to 50 mM NaF for 25 h (*P* < 0.05) (Fig. 6C). These results indicate that heterologous expression of *CsALMT6* enhances tolerance to F toxicity in transgenic yeast.

### Ectopic expression of *CsALMT6* contributes to F toxicity tolerance in transgenic *A. thaliana*

**Figure 7:**
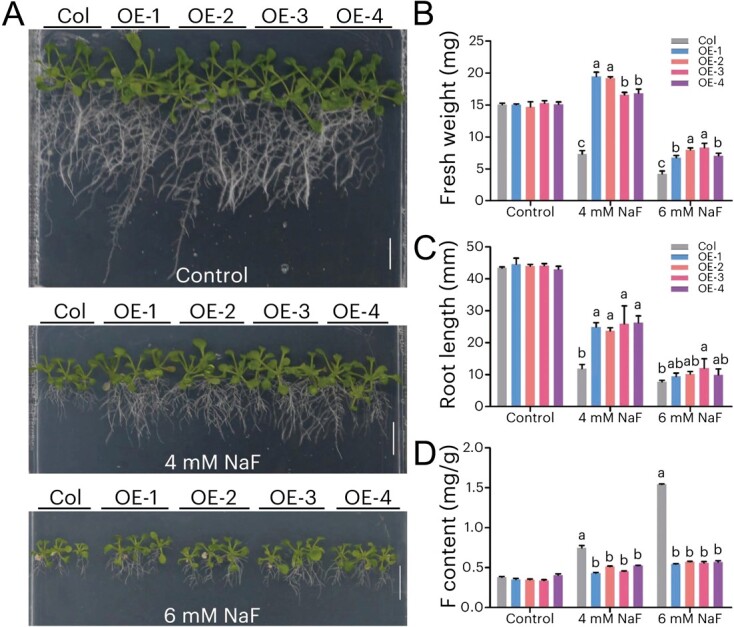
F tolerance of *CsALMT6*-overexpressing *A. thaliana* plants. (A) Phenotypes of *A. thaliana* Columbia ecotype (i.e. Col) and *CsALMT6* transgenic lines (i.e. OE-1, OE-2, OE-3, and OE-4) after exposure to 0 (Control), 4 and 6 mM NaF for 15 days. Bars = 1 cm. (B) Fresh weight, (C) root length, and (D) F content between the Col and *CsALMT6*-overexpressing lines under 0, 4, and 6 mM NaF for 15 days. Data are presented as means ± SD (*n* = 30). Different letters indicate statistically significant differences between the Col and *CsALMT6*-overexpressing lines at *P* < 0.05 under the same F concentration.

To investigate the biological function of *CsALMT6 in planta*, the floral dipping method was used to infect *A. thaliana* Columbia ecotype (Col), and four independent *CsALMT6*-overexpressing lines (i.e. OE-1, OE-2, OE-3, and OE-4) were confirmed using RT-PCR (Supplementary Fig. S4). Subsequently, their seeds were cultivated in the 1/2 Murashige and Skoog (MS) medium treated with different concentrations of NaF [i.e. 0 (Control), 4, and 6 mM] for 15 days. The phenotypes of Col and *CsALMT6* transgenic lines exhibited no significant difference in the absence of F addition (i.e. Control) (Fig. 7). Increasing F stress (i.e. 4 and 6 mM NaF treatments) dramatically inhibited the fresh weight and root growth in both Col and *CsALMT6*-overexpressing lines, resulting in a decrease in fresh weight and root length (Fig. 7A-C). Furthermore, under F-stressed conditions, the fresh weight and root length of *CsALMT6*-overexpressing lines were significantly higher than those of Col (*P* < 0.05). Additionally, when exposed to F-stressed conditions, the F content in *CsALMT6* transgenic lines was significantly lower than that of Col (*P* < 0.05) (Fig. 7D). Therefore, it is hypothesized that *CsALMT6* may enhance the F tolerance of transgenic *A. thaliana* by facilitating the efflux of toxic F ions from plant cells.

### CsALMT6 localizes to the plasma membrane in transgenic Arabidopsis roots

The WOLFPSORT software predicted that the CsALMT6 protein was localized in the plasma membrane (Supplementary Table S1). To further validate the subcellular localization of CsALMT6, GFP fluorescence was examined in the roots of five-day-old T_3_ generation 35S::CsALMT6-EGFP transgenic *A. thaliana* roots. As hypothesized, the GFP signal was distributed in the plasma membrane of *CsALMT6* transgenic *A. thaliana* root cells, while no fluorescence signal was detected in wild-type (WT) *A. thaliana* (Supplementary Fig. S5).

### Overexpression of *CsALMT6* decreases F accumulation and alters the partitioning of F in transgenic *Populus*

**Figure 8:**
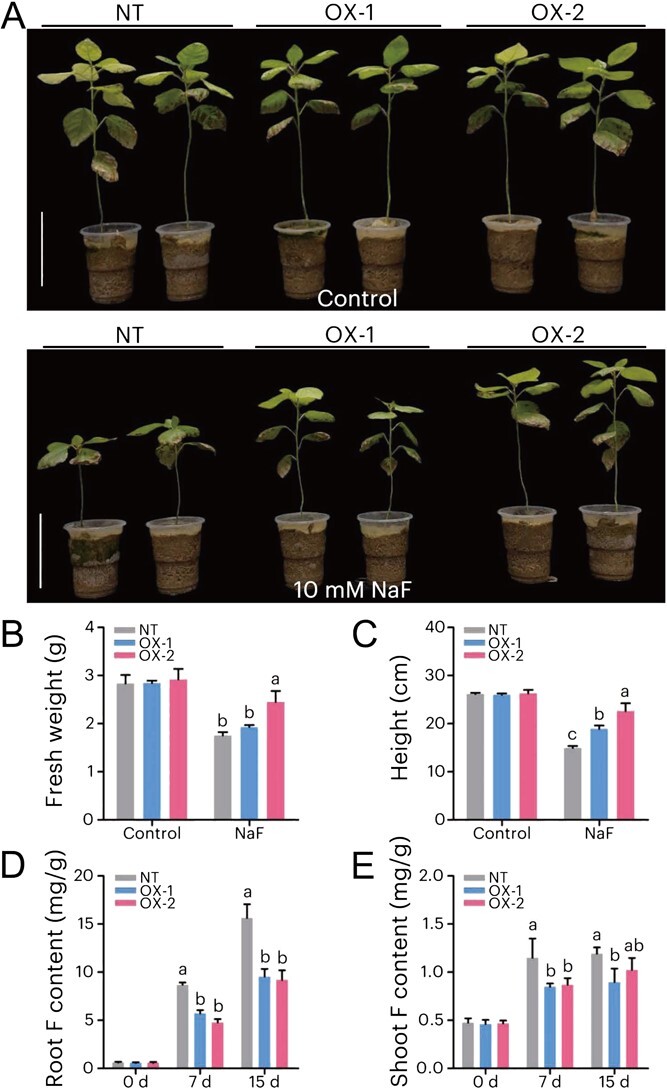
Tolerance of *CsALMT6*-overexpressing *Populus* to F stress. (A) Phenotypes of non-transgenic (i.e. NT) and *CsALMT6* transgenic *Populus* (i.e. OX-1 and OX-2) grown in sand treated with 10 mM NaF for 40 days. Bar = 10 cm. (B) Fresh weight, and (C) height of NT and *CsALMT6*-overexpressing *Populus* exposed to 0 mM (Control) and 10 mM NaF for 40 days. F content in NT and *CsALMT6*-overexpressing *Populus* (D) roots and (E) shoots. Data are expressed the means ± SD (*n* = 4). Different letters indicate significant differences in the NT and *CsALMT6*-overexpressing lines (i.e. OX-1 and OX-2) at *P <* 0.05 under the same treatment conditions.

To further confirm the function of *CsALMT6* in F tolerance, the woody model perennial plant *Populus* was employed, and two *CsALMT6* transgenic lines (i.e. OX-1 and OX-2) were generated and confirmed by RT-PCR (Supplementary Fig. S6). Under normal growth conditions (i.e. Control), the non-transgenic (NT) and *CsALMT6*-overexpressing *Populus* showed no visible differences (Fig. 8A). When treated with 10 mM NaF, the *CsALMT6* transgenic *Populus* exhibited higher fresh weight and height compared to NT (*P* < 0.05) (Fig. 8A-C). Further analysis revealed that F exposure increased the F accumulation in *Populus* root and shoot (Fig. 8D and E). However, the F content in *CsALMT6* transgenic lines was significantly lower than that of NT after F treatment for 7 or 15 days (*P* < 0.05). These results indicated that overexpression of *CsALMT6* confers tolerance to F toxicity by influencing the accumulation and redistribution of F in transgenic *Populus*.

### *CsALMT6* silencing changed the global transcriptome in tea plants

**Figure 9:**
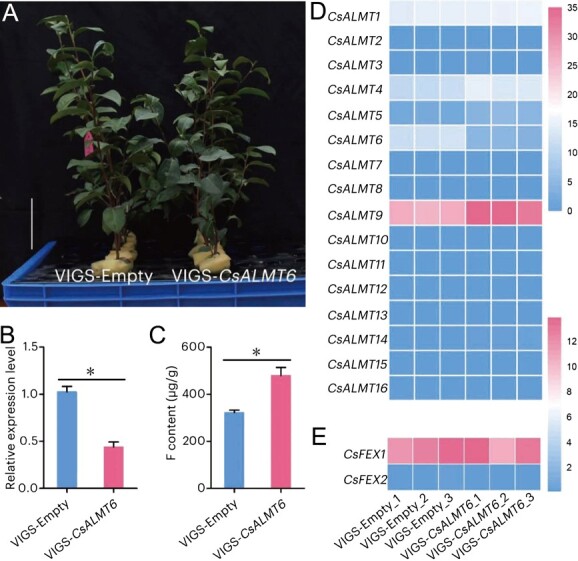
Effect of *CsALMT6* silencing on the accumulation of F in tea plant leaves. (A) Phenotypes of the *CsALMT6*-silenced tea plants. VIGS-Empty, tea plants injected with *Agrobacterium* cells harboring with empty vector pTRV2-C2b. VIGS-*CsALMT6*, tea plants injected with *Agrobacterium* cells harboring with recombinant vector pTRV2-CsALMT6. Bar = 10 cm. (B) The expression level of *CsALMT6* in VIGS-Empty and VIGS-*CsALMT6*. The relative transcription level of *CsALMT6* in VIGS-Empty was set to 1.0, artificially. (C) The content of F in VIGS-Empty and VIGS-*CsALMT6*. The asterisks indicate significant difference at *P* < 0.05. Expression levels of *CsALMT* (D) and *CsFEX* (E) family genes in *CsALMT6*-silenced tea plants based on transcriptome data.

To further verify the function of *CsALMT6* in *C. sinensis*, *CsALMT6*-silenced tea plants were generated and validated by qRT-PCR, which demonstrated a significant down-regulation of *CsALMT6* expression under F-stressed conditions (*P* < 0.05) (Fig. 9A and B). The F content in *CsALMT6*-silenced tea plants (i.e. VIGS-*CsALMT6*) was significantly higher than that in the VIGS-empty tea plants (*P* < 0.05) (Fig. 9C). Transcriptomic analysis revealed that 2438 genes (785 upregulated and 1653 down-regulated) were differentially expressed between VIGS-*CsALMT6* and VIGS-empty (Supplementary Fig. S7). Further analysis of *CsALMT* expression indicated that *CsALMT9* transcript levels were significantly increased in *CsALMT6*-silenced tea plants (*P* < 0.05) (Fig. 9D), suggesting that *CsALMT9* may play a critical role in the response to F toxicity. However, the expression of key genes involved in F tolerance, such as *CsFEX1* and *CsFEX2*, showed no significant change in *CsALMT6*-silenced tea plants (Fig. 9E), indicating that *CsALMT9* may participate in F tolerance through distinct pathways independent of the *CsFEX* modules.

### *CsALMT6* is associated with reducing F accumulation in low-F tea plant cultivar

**Figure 10:**
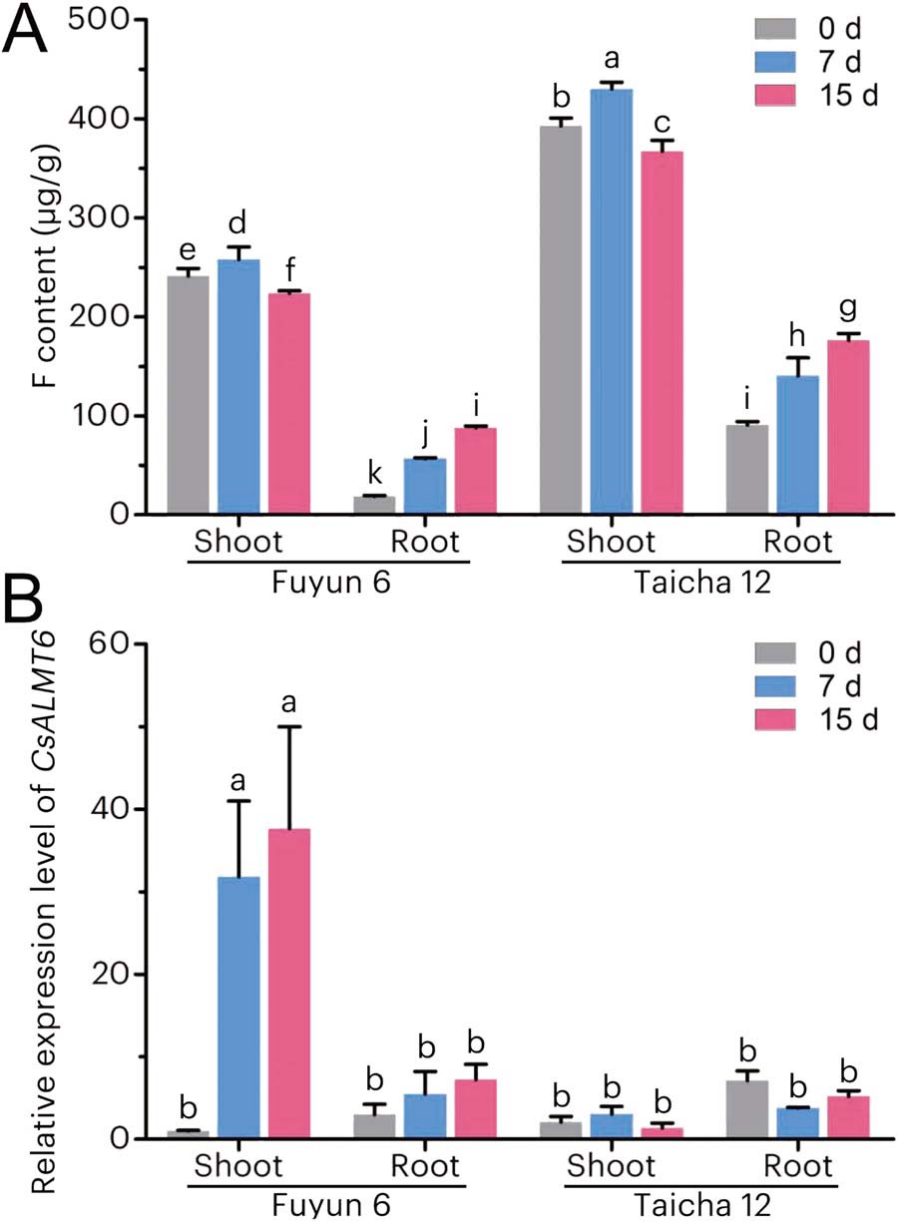
The accumulation of F content (A) and transcriptional profiles of *CsALMT6* (B) in low-F (i.e. 'Fuyun 6′) and high-F (i.e. 'Taicha 12′) tea plant cultivars under F treatments (0.42 mM NaF). The expression levels of *CsALMT6* in the shoot of 'Fuyun 6′ at 0 d were arbitrarily set to 1.0. Data are presented as means ± SD (*n* = 3). Different letters denote statistically significant differences at *P <* 0.05.

Using the F content assessment of 203 tea plant germplasm resources, a low-F tea plant variety ('Fuyun 6′) and a high-F tea plant variety ('Taicha 12′) were randomly selected for further investigation. The objective was to determine whether the functions of *CsALMT6* correlate with the variation in F content among different tea plant cultivars under F toxicity conditions. When subjected to 0.42 mM NaF treatment for 15 days, the F content in each tissue of 'Fuyun 6′ consistently remained lower than that of 'Taicha 12′ at corresponding treatment times (Fig. 10A). Furthermore, the F content exhibited a significant increase in the shoot of both tea cultivars during the initial seven days (*P <* 0.05), followed by a decrease on the 15th day. Moreover, as the NaF treatment duration increased, the F accumulation in the root of both tea cultivars significantly increased (*P <* 0.05), reaching its peak on the 15th day.

Further qRT-PCR analysis indicated that the expression of *CsALMT6* was significantly induced (*P* < 0.05) in both the shoot and root of the low-F cultivar 'Fuyun 6′ under F stress, particularly in the shoot (Fig. 10B). Conversely, when exposed to F stress, the expression of *CsALMT6* in the high-F cultivar 'Taicha 12′ was slightly upregulated in the shoot and down-regulated in the root. Consequently, we hypothesized that *CsALMT6* likely plays a crucial role in F tolerance by reducing F accumulation in the shoot of the low-F cultivar.

## Discussion

GWAS has emerged as a powerful tool for determining candidate genes underlying particular traits and unraveling intricate phenotype–genotype associations [33]. GWAS is widely applied to investigate genetic variation in flora [34–36]. In *C. sinensis*, GWAS has been successfully used to investigate the traits for spring bud flush, leaf shape development, flavor-related metabolites, and other characteristics [37]. However, complex traits are affected by a set of interrelated genes, and GWAS focuses on a single genetic variant with a trait at a time, which may limit the power to identify biologically significant genetic associations [38]. Thus, TWAS serves as a necessary supplement in the identification of candidate genes for complex traits [39], such as growth allometry, metabolite production, and flowering time [40]. In rice, TWAS analyses were employed to identify candidate genes associated with panicle traits [41]. Similarly, BnPMT6 was identified as a negative regulator for seed oil content in *Brassica napus* [42]. These reports support the potential value and feasibility of TWAS in plants. In this study, TWAS was used to explore the candidate genes associated with the F content in *C. sinensis*, and three *CsALMT*s (i.e. *CsALMT1*, *CsALMT2*, and *CsALMT6*) were supposed to be responsible for the differential accumulation of F in the 203 tea plant germplasm resources (Fig. 1). However, it remains unclear whether ALMTs are involved in F uptake in plants, which further arouses our interest.

A comprehensive genome-wide analysis identified a total of 16 *CsALMT* genes in the *C. sinensis* genome (Supplementary Table S1). Phylogenetic analysis categorized these 16 *CsALMT*s into three distinct classes (Fig. 2), a pattern consistent with the findings in *A. thaliana* [43]. Notably, *CsALMT6* formed a separate branch within the *C. sinensis* phylogeny (Fig. 2), indicating a potentially unique function compared to other members of subfamily B. Furthermore, both *CsALMT6* and *CsALMT9* exhibited high expression levels in mature and old leaves (Fig. 3), suggesting their potential involvement in the process of F accumulation in tea plants. Prior research has established that F content varies among different *C. sinensis* cultivars, even under identical growth conditions, with genotype being a primary determinant of F accumulation. To further investigate the expression of *CsALMT*s in response to F stress, this study selected two *C. sinensis* cultivars: 'Longjing 43′, a low-F cultivar, and 'Echa 10′, a high-F cultivar (Fig. 4). Interestingly, the expression levels of *CsALMT6* were significantly upregulated under F-stressed conditions in both cultivars (Fig. 5). Tea plants primarily accumulate excessive F in their leaves without exhibiting symptoms of toxicity, indicating that they may possess mechanisms to either sequester or export F ions [28, 31]. Based on these findings, we hypothesize that *CsALMT6* plays a critical role in F tolerance or accumulation in tea plants.

Plants have evolved various strategies for coping with adverse environmental conditions, and ALMTs play crucial roles in tolerance to Al^3+^ toxicity [44–46]. However, 'Al activation' has only been reported in a small subset of ALMTs. In plants, the functions of a large number of ALMT members have been shown to be highly diverse and transcend beyond root Al-resistance responses [11]. For example, AtALMT9 is known as a Cl^−^ channel [17], and ZmALMT1/2 are permeable for physiologically relevant anions Cl^−^, NO_3_^−^, and SO_4_^2−^ [47, 48]. In this study, *CsALMT6* was selected for further functional characterization due to its response to F stresses in tea plants. Preliminary functional analysis showed that the growth of the *CsALMT6* transgenic yeast was faster than that of the control strain when treated with 30 or 50 mM NaF (Fig. 6), indicating that *CsALMT6* was positively related to F tolerance. Further functional analysis indicated that the root length and fresh weight of *CsALMT6*-overexpressing lines were significantly higher than those of Col when exposed to F-stressed conditions (Fig. 7A-C)*.* Furthermore, *CsALMT6* overexpression reduced the accumulation of F in transgenic *Arabidopsis* compared to the nontransgenic seedling under F toxicity conditions (Fig. 7D). This is the first time that an ALMT family member has been demonstrated to be involved in F tolerance in flora. Previous studies have shown that ALMTs are mostly located in the plasma membrane or vacuole membrane [47, 49]. Here, it was observed that *CsALMT6* was located in the plasma membrane (Supplementary Fig. S5). Combining these results, it is speculated that overexpression of *CsALMT6* contributes to relieving F toxicity by alleviating F accumulation in transgenic *A. thaliana*.

As *C. sinensis* is a perennial woody plant and no stable genetic transformation system has been established until recently, we utilized the woody model plant *Populus* to further investigate the function of *CsALMT6*. Consistent with the results in *CsALMT6* transgenic *A. thaliana*, heterologous expression of *CsALMT6* enhanced tolerance to F toxicity in transgenic *Populus* by reducing the accumulation of F in poplar shoots and roots (Fig. 8). Collectively, these findings provide evidence that *CsALMT6* is involved in detoxifying F through the efflux of excessive F ions from plants.

To further verify the function of *CsALMT6* in *C. sinensis*, *CsALMT6*-silenced tea plants were generated (Fig. 9A and B), in which the content of F was significantly higher than that in the VIGS-Empty tea plants (*P* < 0.05) (Fig. 9C). Interestingly, under F toxicity conditions, the expression of *CsALMT9* was significantly upregulated in *CsALMT6*-silenced plants. This suggests that *CsALMT9* may also contribute to F tolerance in tea plants by maintaining F homeostasis under stress, although further confirmation is required. However, the transcription of key F-tolerant genes, such as *CsFEX1* and *CsFEX2*, exhibited no significant change (Fig. 9D and E), indicating that the *CsALMT6*/*9*-mediated F tolerance pathways may operate independently from the *CsFEX*s modules in tea plants. It is well established that the genotype of tea plants significantly influences their ability to accumulate F [24]. Therefore, an association study between *CsALMT6* expression and F accumulation was conducted using a low-F tea plant cultivar, 'Fuyun 6′, and a high-F tea plant cultivar, 'Taicha 12′. As anticipated, under F-stressed conditions, the transcription of *CsALMT6* was significantly induced in the shoot of the low-F tea cultivar, which exhibited a lower F accumulation level compared to the high-F tea cultivar (Fig. 10). These findings support the speculation that *CsALMT6* plays a role in reducing F accumulation in low-F tea plants.

## Conclusion

**Figure 11:**
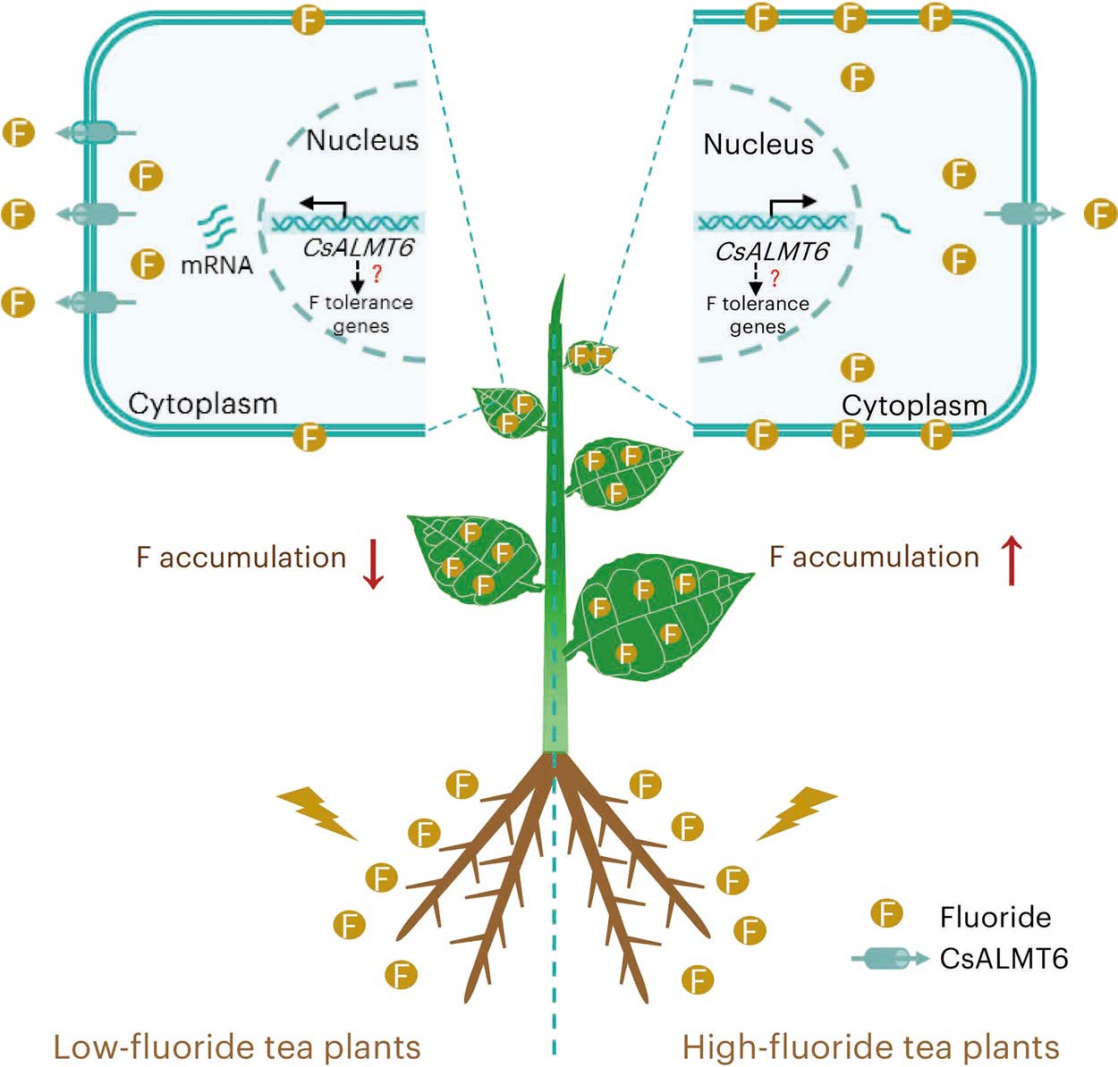
A proposed model for *CsALMT6* in response to F stress in tea plants.

This study utilized TWAS and genome-wide identification of the *CsALMT* family to identify *CsALMT6* as a candidate gene for F tolerance in *C. sinensis*. Ectopic expression of *CsALMT6* in yeast, *Arabidopsis*, and *Populus* confirmed its positive regulatory role in F tolerance within these genetically modified organisms. Notably, the expression of *CsFEX*s remained unaffected in *CsALMT6*-silenced tea plants. Based on these findings, we propose a model (Fig. 11) to explain the role of *CsALMT6* in F toxicity tolerance in tea plants. Under F-stressed conditions, *CsALMT6* transcription is activated, particularly in low-F tea plant cultivars. Subsequently, excessive F concentrations are exported out of cells, facilitating detoxification and reducing F accumulation in tea plants. However, the downstream genes regulated by *CsALMT6* remain unclear. In conclusion, our findings reveal the crucial role of *CsALMT6* in F tolerance in tea plants, expanding our understanding of the functional characterization of ALMTs in plants and highlighting *CsALMT6* as a valuable genetic resource for breeding potentially low-F crops.

## Materials and methods

### Plant materials and growth conditions

One-year-old cuttings of two *C. sinensis* cultivars (i.e. 'Longjing 43′ and 'Echa 10′) were obtained from Lichuang Biotechnology Co., Ltd. (Yichang, Hubei Province, China) and cultivated hydroponically on 20 September 2021. These seedlings were grown hydroponically in an illumination incubator under controlled conditions (24/20°C, 14 h light/10 h dark; 200 *μ*mol·m^−2^·s^−1^; 70% relative humidity). The nutrient solution [50] was renewed every seven days until a substantial number of new adventitious roots developed. Subsequently, the seedlings of the two *C. sinensis* cultivars were treated with 0.42 mM NaF, and the shoots (one bud with two leaves) were harvested at 0, 1, 4, and 12 h, respectively. The harvested samples were immediately frozen in liquid nitrogen and stored at −70°C for qRT-PCR assay. For the VIGS assay, the tea plant cultivar 'Echa 10′ was employed and precultured as described above. All experiments were conducted with three biological replicates.

For the F content trait collection, leaves attached to the green stem were collected from 203 tea plant cultivars grown in the Tea Germplasm Resources Nursery of Huazhong Agricultural University (Wuhan, China) on 8 or 9 October 2021. The F content in different leaf positions (i.e. the bud, first, second, third, fourth, fifth, sixth, and old leaf) of two *C. sinensis* cultivars ('Longjing 43′ and 'Echa 10′) was measured by collecting samples from the Southern Lake Tea Plantation at Huazhong Agricultural University (Wuhan, China) on 6 October 2022. Each sample was collected from twenty tea plants, and each experiment was performed with five biological replicates. Additionally, one-year-old cutting seedlings of tea plant cultivars ('Fuyun 6′, 'Taicha 12′, and 'Fuding Dabaicha') were purchased from Linong Specialized Tea Seedlings Cooperatives (Fu'an City, Fujian Province, China) on 8 September 2023, and precultured as described above. These seedlings were supplied with 0.42 mM NaF on 4 April 2024, and the samples (shoots and roots) of each tea plant cultivar were harvested at 0, 7, and 15 days for F content and qRT-PCR assays. Furthermore, samples of 'Fuding Dabaicha' from different leaf positions [i.e. bud, first leaf, first MV, third leaf, third MV, fifth leaf, fifth MV], stem, and root were harvested for gene expression analysis. These experiments were conducted with three biological replicates.

### TWAS analysis

Raw transcriptome data were downloaded [51], and high-quality clean reads were mapped to the *C. sinensis* cv. 'Shuachazao' reference genome [52] using HISAT2 [52]. GATK (v3.8) was employed to identify single-base mismatches as potential single nucleotide polymorphisms (SNP) sites. After filtering according to minor allele frequency (MAF: 0.01) and site completeness (INT: 0.8), a highly consistent set of 964 798 SNP sites was obtained for downstream analysis. Association analyses of SNPs were performed using the GLM in TASSEL software (v5.2.34) based on a panel of 203 samples collected from the NCBI Database.

### Genome-wide identification of *CsALMT*s family members from *C. sinensis*

To identify all *CsALMT* family members from *C. sinensis*, we obtained the genomic dataset of *C. sinensis* 'Shuchazao', which includes amino acid sequences, generic feature format version 3 (GFF3), and Pfam domains data [53]. We also acquired Hidden Markov model (HMM) profiles of the ALMT domain (PF11744) and employed HMM searches using HMMER software (v3.0) to identify *ALMT* genes in the tea plant genome [54]. ALMTs lacking conserved domains were excluded from the analysis [55]. Finally, the coding sequence of each putative *CsALMT* gene was amplified and sequenced to validate the results of the bioinformatics analyses. The primers are listed in Supplementary Table S4.

### Bioinformatics analyses of *CsALMT*s

The physicochemical parameters of CsALMT proteins were determined using the ExPASy program (http://web.expasy.org/compute_pi/). Subcellular localization of CsALMTs was predicted using WoLF PSORT [56]. Phylogenetic trees were constructed using the neighbor-joining method by MEGA software [57], with a bootstrap test replicate set to 1000. The chromosomal locations and structures of *CsALMT*s were visualized employing TBtools software [58]. The MEME (http://meme-suite.org/tools/meme) and WebLogo (http://weblogo.berkeley.edu/logo.cgi) programs were used to analyze and illustrate the conserved motifs of *CsALMT*s [59, 60].

### Transcriptional analyses of *CsALMT*s

To investigate the transcription profiles of *CsALMT*s across various tissues, the transcript per million (TPM) values of *CsALMT*s in eight tissues (apical bud, young leaf, flower, mature leaf, fruit, old leaf, stem, and root) of *C. sinensis* were obtained from the Tea Plant Information Archive [53].

To determine the F stress response of *CsALMT*s, a quantitative reverse transcription polymerase chain reaction (qRT-PCR) assay was performed according to the method described in TB Green^®^*Premix Ex Taq*™ II (Tli RNaseH Plus) (Takara, Beijing, China). The relative transcription levels of each *CsALMT* were calculated using the 2^–ΔΔCT^ method [61] and normalized to four housekeeping genes (*CsEF-1α*, *CsTIP41*, *CsTBP*, and *CsACTIN*) in *C. sinensis*. The expression profiles of *CsALMT*s were visualized using the HemI software [62]. The primers are listed in Supplementary Table S4.

### F content analysis

To determine the F content, the samples were initially dried in an oven at 105°C for ten minutes and further dried at 75°C until a constant mass was achieved. Following drying, the samples were pretreated according to the procedure previously described [63]. Briefly, each sample underwent digestion with NaOH, and the analyte was subsequently mixed in a beaker with total ionic strength adjustment buffers (TISABs). The F content of the samples was then determined using Thermo Scientific^™^ Orion^™^ Fluoride Electrodes.

### F tolerance analyses of *CsALMT6* in yeast

The open reading frame (ORF) of *CsALMT6* with stop codons was amplified with pYES2-CsALMT6-F and pYES2-CsALMT6-R (Supplementary Table S4) and inserted into the *Kpn*I and *Bam*HI sites of the pYES2 vector. Subsequently, the fusion vector (i.e. pYES2-*CsALMT6*) and the empty vector pYES2 were transformed into *S. cerevisiae* INVSc1 competent cells, respectively.

For the F tolerance assay, the transgenic yeast cells (i.e. CsALMT6 and vector) were individually precultured in the YPDA liquid medium until the optical density at 600 nm (OD_600_) reached 0.8. Subsequently, the 10-fold diluted yeast cells were spotted on YPDA medium plates supplemented with 0, 30, and 50 mM NaF, respectively. Representative photographs were taken after cultivation at 30°C in the dark for two days. Additionally, growth curves were determined in YPDA liquid medium with or without 50 mM NaF, and the OD_600_ was recorded for 25 hours. Each sample had four biological replicates.

### F tolerance analyses of *CsALMT6* in *A. thaliana*

The coding sequence of *CsALMT6* was amplified without stop codons using primers 2300-CsALMT6-F and 2300-CsALMT6-R (Supplementary Table S4). Subsequently, the amplification product was inserted into pCAMBIA2300-C-EGFP at the *Kpn*I and *Bam*HI sites. Following sequence confirmation, the recombinant vector 35S::CsALMT6-EGFP was introduced into *Agrobacterium tumefaciens* GV3101 competent cells and transformed into *A. thaliana* [64]. Homozygous T_3_ transgenic Arabidopsis lines were selected using 1/2 MS solid medium supplemented with 50 *μ*g·mL^−1^ kanamycin.

To assess F tolerance, seeds from the Col and transgenic *A. thaliana* lines (OE-1, OE-2, OE-3, and OE-4) were sown on 1/2 MS solid medium supplemented with 0, 4, or 6 mM NaF. Following 15 days of cultivation, the phenotypes of the *A. thaliana* seedlings were observed and representative samples were photographed. Additionally, root length and fresh weight measurements were recorded for each *A. thaliana* plant. The F content in each transgenic Arabidopsis line was determined using the aforementioned methods. All treatments included a minimum of thirty seedlings and were conducted in triplicate.

### Subcellular localization of CsALMT6

To investigate the subcellular localization of *CsALMT6*, the roots of five-day-old T_3_ generation *CsALMT6* transgenic *A. thaliana* plants were examined for GFP fluorescence using a Leica DM5500 B confocal laser-scanning microscope (Leica, Wetzlar, Germany). A minimum of six individual transgenic plants were utilized to observe the GFP signal, and the representative images were presented with the WT Arabidopsis serving as a negative control.

### F tolerance analyses of *CsALMT6* in *Populus*

The *Populus* line NL895 (*Populus deltoides* × *Populus euramericana* cv. 'Nanlin 895′) was employed to generate *CsALMT6* transgenic *Populus* according to the methods described in previous study [65]. Briefly, *Populus* leaves were utilized to conduct tissue culture with *A. tumefaciens* GV3101 cells harboring 35S::CsALMT6-EGFP. Subsequently, the callus was induced to produce buds in the bud induction medium containing 100 mg·L^−1^ kanamycin, followed by PCR amplification with *CsALMT6* gene-specific primers (*CsALMT6*-F and *CsALMT6*-R) to confirm the positive *CsALMT6*-overexpressing lines, in which *PdeACTIN* served as an internal reference gene [66] (Supplementary Table S4). All tissue cultures of 'NL895' were conducted in the woody plant medium (WPM) at 22°C with a light intensity of 130 μmol·m^−2^·s^−1^ and a 16/8 h photoperiod.

To investigate the F tolerance of *CsALMT6*, microcuttings of non-transgenic (i.e. NT) and *CsALMT6*-overexpressing *Populus* (i.e. OX-1 and OX-2) were propagated for 35 days in the WPM. Subsequently, these *Populus* were transferred to pots filled with sand and treated with Hoagland solution (with or without 10 mM NaF) every five days. After cultivation under normal growth conditions for 40 days, the phenotypes of these *Populus* were photographed, and the fresh weight and shoot height were recorded. Furthermore, after exposure to NaF treatments (i.e. 10 mM) for 7 and 15 days, the F content in *Populus* roots and shoots was measured as described above. Each experiment was repeated with four biological replicates.

### VIGS of *CsALMT6* in tea plants

To further investigate the function of *CsALMT6* in tea plants, VIGS technology was employed to silence the expression of *CsALMT6* following a previously reported method [67]. A gene-specific fragment of *CsALMT6* (250 base pairs in length) was amplified with pTRV2-CsALMT6-F and pTRV2-CsALMT6-R primers (Supplementary Table S4) and inserted into the *Sma*I site of the pTRV2-C2b vector to construct the pTRV2-CsALMT6 recombinant vector. The pTRV1, pTRV2-C2b, and pTRV2-CsALMT6 vectors were introduced into the *Agrobacterium* GV3101 competent cells [68], respectively. A mixture of pTRV1 and pTRV2-CsALMT6 (OD_600_ = 1.2) *A. tumefaciens* in a ratio of 1:1 (v:v) was injected into the leaves of hydroponic-cultured *C. sinensis*. The control group was injected with the mixture of pTRV1 and pTRV2-C2b *Agrobacterium* solution.

The inoculated tea seedlings were maintained in dark conditions with high humidity for three days. Subsequently, they were exposed to a 0.42 mM NaF treatment for 7 days under standard growth conditions. Following the treatment, samples were collected for qRT-PCR and RNA-sequencing analyses. To ensure reliability, each experiment was conducted with three biological replicates.

### Transcriptomic analysis

Tea leaves with silenced *CsALMT6* expression were subjected to transcriptome analyses, with tea leaves injected with pTRV1 and pTRV2-C2b *Agrobacterium* (VIGS-Empty) serving as a control. The RNA-sequencing protocol was as follows: (i) RNA extraction was performed using the Quick RNA Isolation Kit (Huayueyang, Beijing, China), and quality assessment was conducted using a Bioanalyzer 2100 System (Agilent Technologies, Santa Clara, CA, USA); (ii) the purified RNA samples were submitted to Tsingke Biotech Co., Ltd. (Beijing, China) for RNA-sequencing, and the clean reads were aligned to the *C. sinensis* reference genome sequence [69] using HISAT2 and StringTie software, and (iii) functional annotation of the unigenes was performed using public databases, and the expression values of all genes were normalized to fragments per kilobase of TPM fragments mapped (FPKM). The experiment was conducted with three biological replicates.

### Statistical analyses

Data analyses were conducted using IBM SPSS Software (v25.0) (IBM Corp., Armonk, NY, USA). Data are presented as means ± SD. Analysis of variance (ANOVA) and Duncan's test were employed to determine significant differences between the experimental data.

## Supplementary Material

Web_Material_uhae353

## Data Availability

All relevant data in this study are provided in the article and its supplementary file.
