# Supplementary material for: Aluminum-activated malate transporter family member CsALMT6 mediates fluoride resistance in tea plants (Camellia sinensis)
Source: Hortic Res. 2024 Dec 12;12(4):uhae353. doi: 10.1093/hr/uhae353 (PMC11879333; doi:10.1093/hr/uhae353)
Supplement: Web_Material_uhae353 [file web_material_uhae353.zip › Supplementary Tables.docx]

**Table S1.** Characteristics of *CsALMT* family genes

| Gene name | Genome ID | ORF (bp) | Amino acids (aa) | MW (kDa) | pI | GRAVY | Subcellular localization |
| --- | --- | --- | --- | --- | --- | --- | --- |
| CsALMT1 | TEA013305.1 | 1401 | 466 | 52.04 | 8.13 | 0.070 | plasma membrane |
| CsALMT2 | TEA013312.1 | 1308 | 435 | 47.81 | 5.98 | 0.021 | plasma membrane |
| CsALMT3 | TEA018561.1 | 1272 | 423 | 46.02 | 6.96 | 0.202 | plasma membrane |
| CsALMT4 | TEA000542.1 | 2382 | 793 | 88.72 | 6.38 | –0.036 | plasma membrane |
| CsALMT5 | TEA002026.1 | 1761 | 586 | 65.65 | 5.66 | ‒0.009 | plasma membrane |
| CsALMT6 | TEA032422.1 | 1614 | 537 | 60.07 | 6.26 | 0.003 | plasma membrane |
| CsALMT7 | TEA013308.1 | 1524 | 507 | 56.76 | 8.61 | 0.059 | plasma membrane |
| CsALMT8 | TEA020925.1 | 1470 | 489 | 53.55 | 8.02 | 0.001 | plasma membrane |
| CsALMT9 | TEA032049.1 | 1806 | 601 | 68.68 | 5.96 | ‒0.152 | plasma membrane |
| CsALMT10 | TEA003844.1 | 1464 | 487 | 54.42 | 5.72 | 0.034 | plasma membrane |
| CsALMT11 | TEA027338.1 | 1479 | 492 | 54.83 | 7.03 | 0.070 | plasma membrane |
| CsALMT12 | TEA010694.1 | 1473 | 490 | 54.67 | 6.53 | 0.067 | plasma membrane |
| CsALMT13 | TEA010693.1 | 1227 | 408 | 45.85 | 6.11 | 0.055 | plasma membrane |
| CsALMT14 | TEA025995.1 | 1503 | 500 | 55.84 | 8.98 | 0.038 | plasma membrane |
| CsALMT15 | TEA016261.1 | 1560 | 519 | 58.11 | 8.97 | ‒0.041 | plasma membrane |
| CsALMT16 | TEA029916.1 | 1257 | 418 | 46.29 | 7.08 | 0.215 | plasma membrane |

Note: GRAVY stands for grand average of hydropathicity.

Table S2. The transcript per million (TPM) values of CsALMTs in the eight tissues of C. sinensis

| Gene name | Apical bud | Young leaf | Mature leaf | Old leaf | Stem | Flower | Fruit | Root |
| --- | --- | --- | --- | --- | --- | --- | --- | --- |
| CsALMT1 | 12.14 | 9.24 | 9.61 | 7.27 | 12.24 | 22.97 | 4.56 | 49.47 |
| CsALMT2 | 4.41 | 2.49 | 3.53 | 4.56 | 5.29 | 5.57 | 3.13 | 59.07 |
| CsALMT3 | 0.25 | 0.00 | 0.19 | 1.16 | 1.20 | 1.06 | 5.43 | 8.84 |
| CsALMT4 | 6.61 | 6.39 | 7.80 | 7.70 | 12.59 | 11.72 | 8.79 | 9.14 |
| CsALMT5 | 12.12 | 5.65 | 6.43 | 2.36 | 16.56 | 7.26 | 22.13 | 4.59 |
| CsALMT6 | 0.41 | 0.35 | 11.49 | 11.53 | 3.24 | 4.49 | 0.07 | 0.47 |
| CsALMT7 | 0.00 | 0.00 | 0.08 | 0.03 | 3.13 | 0.00 | 0.05 | 1.21 |
| CsALMT8 | 0.83 | 0.32 | 0.03 | 0.00 | 4.71 | 0.38 | 0.03 | 1.39 |
| CsALMT9 | 26.36 | 25.85 | 27.05 | 30.50 | 27.55 | 25.39 | 31.86 | 2.50 |
| CsALMT10 | 3.70 | 1.01 | 0.00 | 0.00 | 2.88 | 0.95 | 0.16 | 3.14 |
| CsALMT11 | 0.04 | 0.00 | 0.08 | 0.00 | 0.00 | 0.02 | 0.00 | 16.69 |
| CsALMT12 | 2.19 | 0.08 | 0.00 | 0.00 | 0.27 | 0.07 | 0.00 | 5.41 |
| CsALMT13 | 0.00 | 0.00 | 0.00 | 0.00 | 0.00 | 0.00 | 0.00 | 0.00 |
| CsALMT14 | 0.04 | 0.76 | 0.78 | 2.52 | 0.00 | 5.44 | 0.03 | 0.22 |
| CsALMT15 | 1.30 | 3.11 | 0.26 | 0.03 | 3.30 | 0.13 | 0.69 | 0.30 |
| CsALMT16 | 0.19 | 0.33 | 0.27 | 0.17 | 0.10 | 0.25 | 0.35 | 0.26 |

Table S3. The expression levels of CsALMTs in C. sinensis shoots exposed to F treatments (i.e., 0.42 mM NaF)

| Gene name | Relative expression levels | | | | | | | | |  | Log2 (fold changes) | | | | | | | | |
| --- | --- | --- | --- | --- | --- | --- | --- | --- | --- | --- | --- | --- | --- | --- | --- | --- | --- | --- | --- |
|  | Longjing 43 | | | |  | Echa 10 | | | |  | Longjing 43 | | | |  | Echa 10 | | | |
|  | 0 h | 1 h | 4 h | 12 h |  | 0 h | 1 h | 4 h | 12 h |  | 0 h | 1 h | 4 h | 12 h |  | 0 h | 1 h | 4 h | 12 h |
| *CsALMT1* | 1.00 | 1.91 | 0.91 | 1.68 |  | 1.00 | 1.10 | 2.71 | 1.37 |  | 0.00 | 0.93 | -0.14 | 0.75 |  | 0.00 | 0.13 | 1.44 | 0.45 |
| *CsALMT2* | 1.00 | 0.23 | n.d. | 0.42 |  | n.d. | n.d. | n.d. | n.d. |  | 0.00 | -2.12 | n.d. | -1.25 |  | n.d. | n.d. | n.d. | n.d. |
| *CsALMT3* | 1.00 | 0.32 | 2.42 | 0.59 |  | 1.00 | n.d. | 0.21 | n.d. |  | 0.00 | -1.64 | 1.28 | -0.77 |  | 0.00 | n.d. | -2.22 | n.d. |
| *CsALMT4* | 1.00 | 1.11 | 1.05 | 1.10 |  | 1.00 | 1.45 | 1.10 | 1.14 |  | 0.00 | 0.16 | 0.07 | 0.14 |  | 0.00 | 0.54 | 0.14 | 0.19 |
| *CsALMT5* | 1.00 | 2.93 | 1.01 | 0.46 |  | 1.00 | 3.28 | 0.56 | 0.45 |  | 0.00 | 1.55 | 0.01 | -1.11 |  | 0.00 | 1.71 | -0.83 | -1.14 |
| *CsALMT6* | 1.00 | 12.42 | 18.10 | 5.40 |  | 1.00 | 79.15 | 122.70 | 69.71 |  | 0.00 | 3.63 | 4.18 | 2.43 |  | 0.00 | 6.31 | 6.94 | 6.12 |
| *CsALMT7* | 1.00 | 1.72 | 0.52 | 0.53 |  | 1.00 | 0.07 | 0.10 | 0.04 |  | 0.00 | 0.78 | -0.94 | -0.91 |  | 0.00 | -3.81 | -3.28 | -4.74 |
| *CsALMT8* | n.d. | n.d. | n.d. | n.d. |  | n.d. | n.d. | n.d. | n.d. |  | n.d. | n.d. | n.d. | n.d. |  | n.d. | n.d. | n.d. | n.d. |
| *CsALMT9* | 1.00 | 1.01 | 1.00 | 0.68 |  | 1.00 | 2.22 | 3.02 | 2.30 |  | 0.00 | 0.01 | 0.00 | -0.55 |  | 0.00 | 1.15 | 1.59 | 1.20 |
| *CsALMT10* | n.d. | n.d. | n.d. | n.d. |  | 1.00 | 0.03 | 0.04 | n.d. |  | n.d. | n.d. | n.d. | n.d. |  | 0.00 | -5.12 | -4.67 | n.d. |
| *CsALMT11* | n.d. | n.d. | n.d. | n.d. |  | n.d. | n.d. | n.d. | n.d. |  | n.d. | n.d. | n.d. | n.d. |  | n.d. | n.d. | n.d. | n.d. |
| *CsALMT12* | n.d. | n.d. | n.d. | n.d. |  | n.d. | n.d. | n.d. | n.d. |  | n.d. | n.d. | n.d. | n.d. |  | n.d. | n.d. | n.d. | n.d. |
| *CsALMT13* | n.d. | n.d. | n.d. | n.d. |  | n.d. | n.d. | n.d. | n.d. |  | n.d. | n.d. | n.d. | n.d. |  | n.d. | n.d. | n.d. | n.d. |
| *CsALMT14* | 1.00 | 0.59 | 0.90 | 0.28 |  | 1.00 | 0.37 | 0.34 | 0.12 |  | 0.00 | -0.76 | -0.15 | -1.82 |  | 0.00 | -1.42 | -1.56 | -3.02 |
| *CsALMT15* | 1.00 | n.d. | n.d. | 1.22 |  | 1.00 | n.d. | n.d. | n.d. |  | 0.00 | n.d. | n.d. | 0.29 |  | 0.00 | n.d. | n.d. | n.d. |
| *CsALMT16* | 1.00 | 0.72 | 1.78 | 1.46 |  | 1.00 | 0.03 | 0.07 | 0.07 |  | 0.00 | -0.47 | 0.83 | 0.55 |  | 0.00 | -5.00 | -3.91 | -3.81 |

Note: n.d. indicated the expression was too low to be detected.

Table S4. Primers used in the study

| Primer name | Sequence (5'-3') | Function |
| --- | --- | --- |
| CsALMT1-F  CsALMT1-R | ATGGCAACCCCAAGTAATGAG  TCATTCAGCTGGTCCTTGAAA | Cloning of *CsALMT*s |
| CsALMT2-F  CsALMT2-R | ATGGAAATCGAATCTGAAACCA  TCATTCTCCTGGGCTTTGAATTG |  |
| CsALMT3-F  CsALMT3-R | ATGGGTTCAACTGTGATAAC  CTATCTAGTTTGAAAATCAG |  |
| CsALMT4-F  CsALMT4-R | ATGTACTATCCTTGTGATAGGT  TTAAGCTGAGCTAGTTCCAATT |  |
| CsALMT5-F  CsALMT5-R | ATGGCAACAAAAATTGGTTCATTC  TCAATCATTGAACCAAAAACACTTA |  |
| CsALMT6-F  CsALMT6-R | ATGAATGGAAAAAAGGGTAGTT  TTATTGAACCTCATACTTGAAC |  |
| CsALMT7-F  CsALMT7-R | ATGGAAATTGGGTCTGAAAACT  TTACACTTTCACAAGCTGACTA |  |
| CsALMT8-F  CsALMT8-R | ATGGAACTCGAATCAAAAACCC  TTAAGCTTCAACAGCTCTTTGG |  |
| CsALMT9-F  CsALMT9-R | ATGGAGAGAGAAGAGAGAAAGA  CTATCCCAACAACTGCTCATCT |  |
| CsALMT10-F  CsALMT10-R | ATGAATGCCATAAAGGAAGCTC  TCAGACCTCCTCAAGGACTTTC |  |
| CsALMT11-F  CsALMT11-R | ATGGCTAAAGAACAGAGACTTG  TTATGTTGAACTCAGACCTTTT |  |
| CsALMT12-F  CsALMT12-R | ATGGCTAGTGAAAAAGAATCGTG  TCAGATCTTTTCAAGGATTTTC |  |
| CsALMT13-F  CsALMT13-R | ATGAGACCTTTGTATGATGGTG  TTAGACCTTTTCAAGGATTTTC |  |
| CsALMT14-F  CsALMT14-R | ATGTTTTCTGGCGTTGCTAACG  TTAATCTGCTCCATGGGAAGGC |  |
| CsALMT15-F  CsALMT15-R | ATGGACACAGAAAGCCAGGAGAC  CTAATCTAATCCATGGGAAGCC |  |
| CsALMT16-F  CsALMT16-R | ATGAGTTCAACTGTAATAACTA  TTAGTTTGAAAAACCTGCTAGTG |  |
| CsALMT1-qF  CsALMT1-qR | CAATGGCTGCCTTGACTCTC  CTCCAGGAGGTCTGTGTCAG | qRT-PCR |
| CsALMT2-qF  CsALMT2-qR | CAATGTGCCTGCCGTATTGA  GTGCTCAACTTTGTGCTGGA |  |
| CsALMT3-qF  CsALMT3-qR | GAGAGCTCGGAGAGAGCATT  GAAGGAGAAGCGAGTAGGCT |  |
| CsALMT4-qF  CsALMT4-qR | TCAGAGGGTTGGCTCAGAAG  TCTTCTGCAGCTTCATGCAC |  |
| CsALMT5-qF  CsALMT5-qR | TGCGAGCTCATTGTCATTGG  TCTCTCCCGGTGCAATATCC |  |
| CsALMT6-qF  CsALMT6-qR | GGTCAATCCTCACTGTTGCC  ACGACCCGGAATCCATACTC |  |
| CsALMT7-qF  CsALMT7-qR | TATTGGTGCGACTGCTTGTG  GAAGCTGCCAAGTGTTTCCA |  |
| CsALMT8-qF  CsALMT8-qR | CCTGTATGGGCTGGTGAAGA  GGTTCCCACCATGCAAAGTT |  |
| CsALMT9-qF  CsALMT9-qR | CCTGCAAACTTGTCGTGGAA  GCCGTGCAACGAATTCAATC |  |
| CsALMT10-qF  CsALMT10-qR | AGAAGGCATGGGAGTTAGGG  TCATGATAGCCCACATGGCA |  |
| CsALMT11-qF  CsALMT11-qR | TTTGCTGACCGAAATCGCAG  TCACTCGCAGCCTTGAACTT |  |
| CsALMT12-qF  CsALMT12-qR | GCAGGCAAAGAGCTTCACTT  CATTCGTAACCTCGCAGCAT |  |
| CsALMT13-qF  CsALMT13-qR | ACCGCTTCAACAGCAACTTT  TGCAGATGGTGGTTCCAATG |  |
| CsALMT14-qF  CsALMT14-qR | GGCTTCACTATCAAGCGCAA  CTTGCCACTGTCTCCACAAG |  |
| CsALMT15-qF  CsALMT15-qR | CCTCAAATCCCAACCACGTC  TCACGCTCGATAAGGAGACC |  |
| CsALMT16-qF  CsALMT16-qR | GACAGCCCTCATCAGCTCTA  GCCTTAGGCCTGCATCTTTC |  |
| CsEF-1α-qF  CsEF-1α-qR  CsTIP41-qF  CsTIP41-qR  CsTBP-qF  CsTBP-qR  CsACTIN-qF  CsACTIN-qR | CAAGCGTGTCATCGAGAGAT  ATACCACGTTCACGTTCAGC  CGAAAGAGCCCATTCTCTTC  ACGTGTGTCCCTCAATCTCA  AAGGGATCCAAAGACGACAG  TGAAATCCTTGAATTTGGCA  CAGACCGTATGAGCAAGGAA  GCTTAGGGATGCGAGGATAG | Internal reference genes for *C. sinensis* |
| AtACTIN2-F  AtACTIN2-R | CTCCCGCTATGTATGTCGCC  TTGGCACAGTGTGAGACACAC | Internal reference gene for *A. thaliana* |
| PdeACTIN-F  PdeACTIN-R | TCATCGGAATGGAAGCTGCTGGTA  TAGTGGAACCACCACTGAGCACAA | Internal reference gene for *Populus* |
| pYES2-CsALMT6-F  pYES2-CsALMT6-R | GGAATATTAAGCTTGGTACCATGAATGGAAAAAAGGGTAG  CGGCCGTTACTAGTGGATCCTTATTGAACCTCATACTTGAAC | Construction of yeast expression vector |
| 2300-CsALMT6-F  2300-CsALMT6-R | TTTGGAGAGGACAGGGTACCATGAATGGAAAAAAGGGTAG  CCTCCTCCTCTAGAGGATCCTTGAACCTCATACTTGAAC | Construction of plant expression vector |
| pTRV2-CsALMT6-F  pTRV2-CsALMT6-R | AGGCCCGGTGAGGAGAAGAGCCCGGGATGAATGGAAAAAAGGGTAG  TTGTGTGCTCGACGACAAGACCCGGGTGGTGCCGAACACTTGATAAGG | Silencing of *CsALMT6* using VIGS |
