# Supplementary figures and images for: Aluminum-activated malate transporter family member CsALMT6 mediates fluoride resistance in tea plants (Camellia sinensis)
Source: Hortic Res. 2024 Dec 12;12(4):uhae353. doi: 10.1093/hr/uhae353 (PMC11879333; doi:10.1093/hr/uhae353)

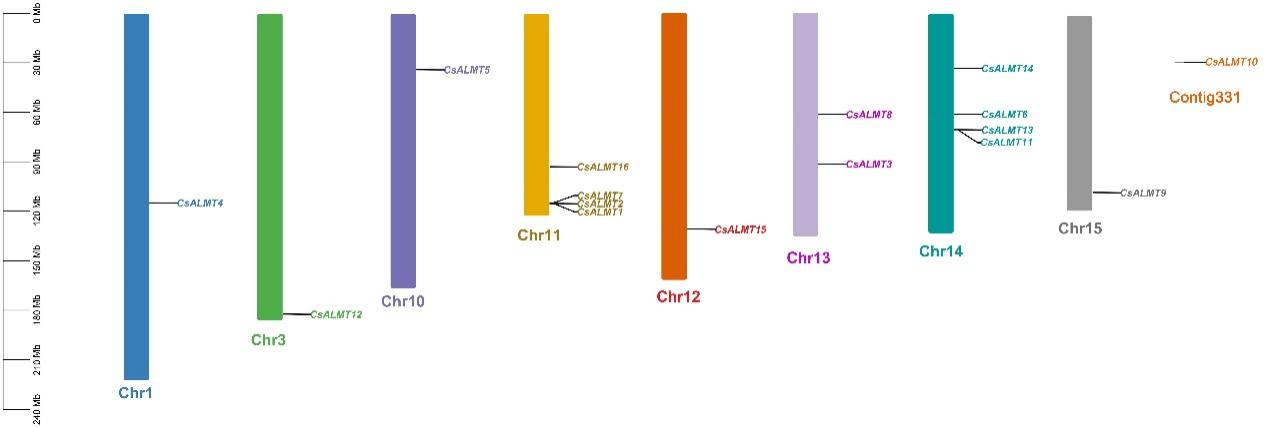


**Fig. S1**


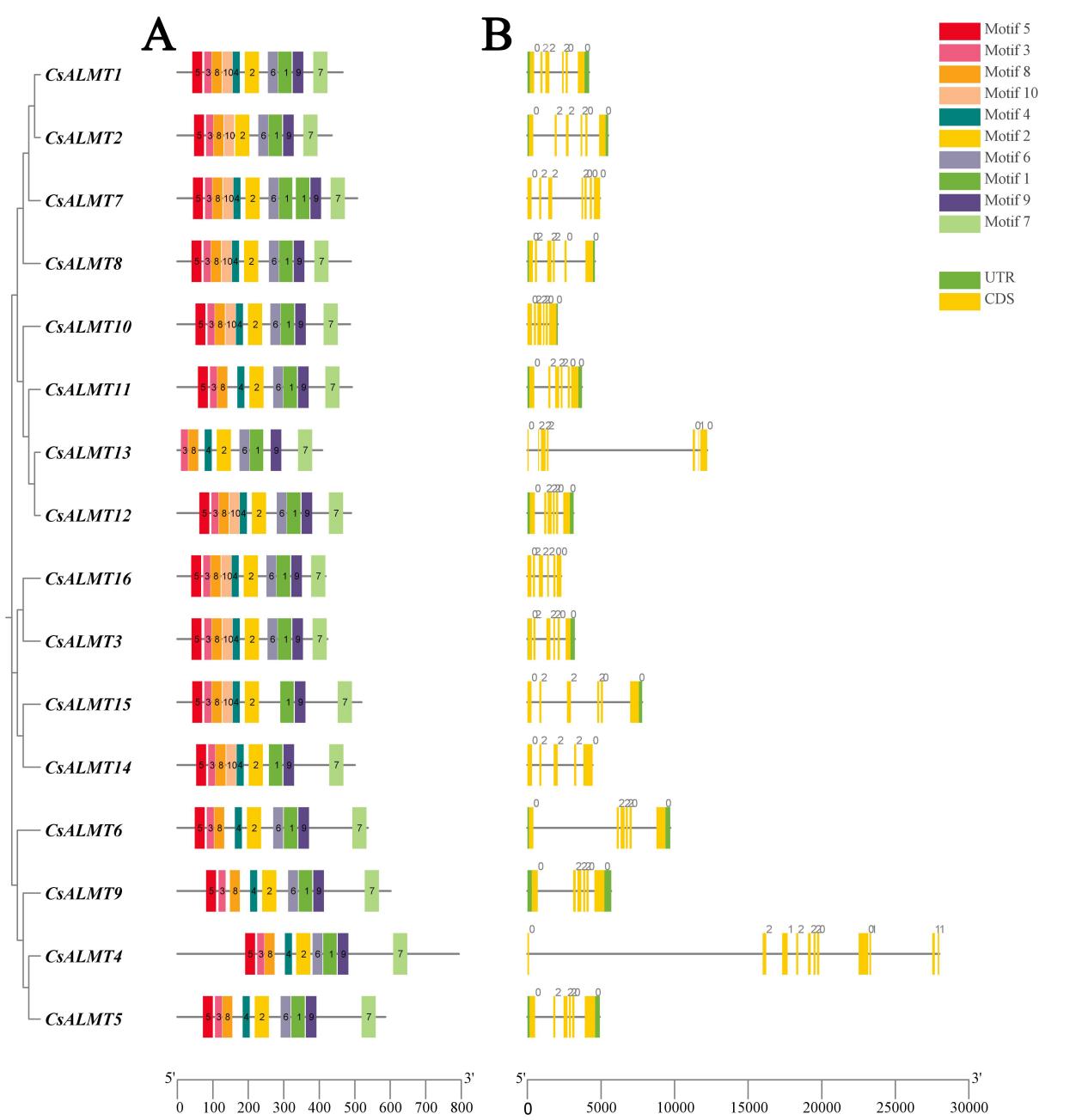


**Fig. S2**


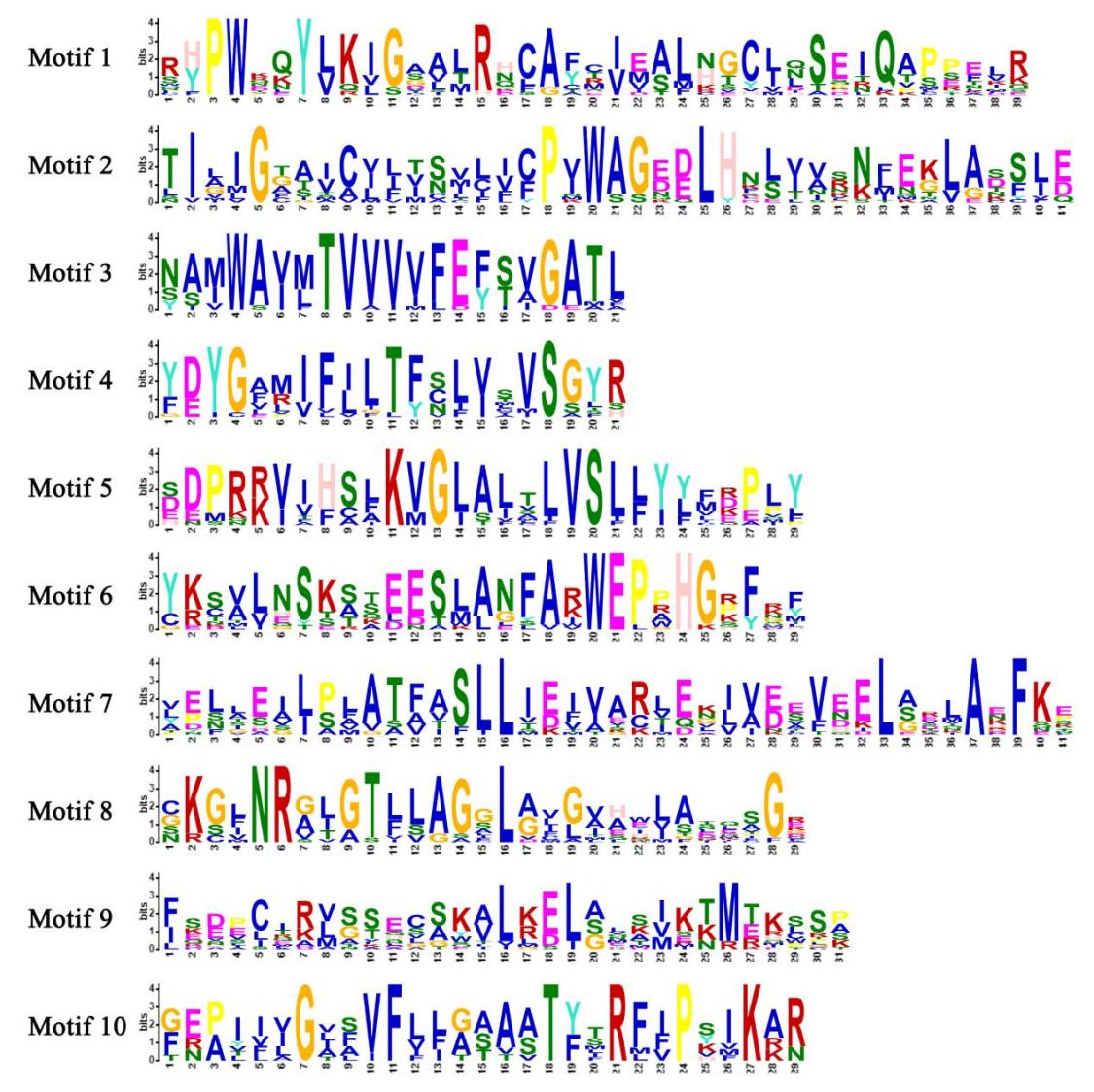


**Fig. S3**


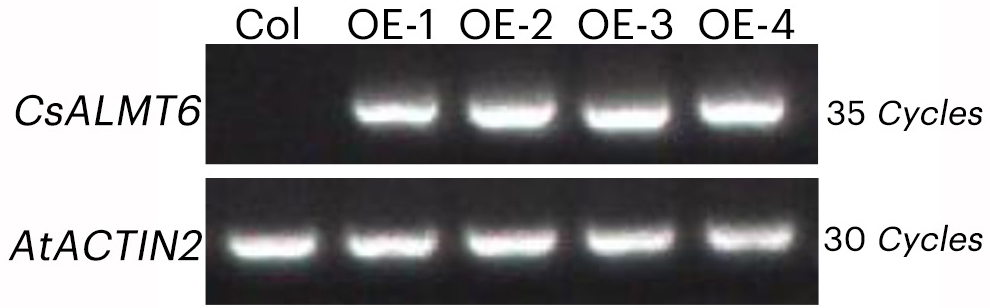


**Fig. S4**


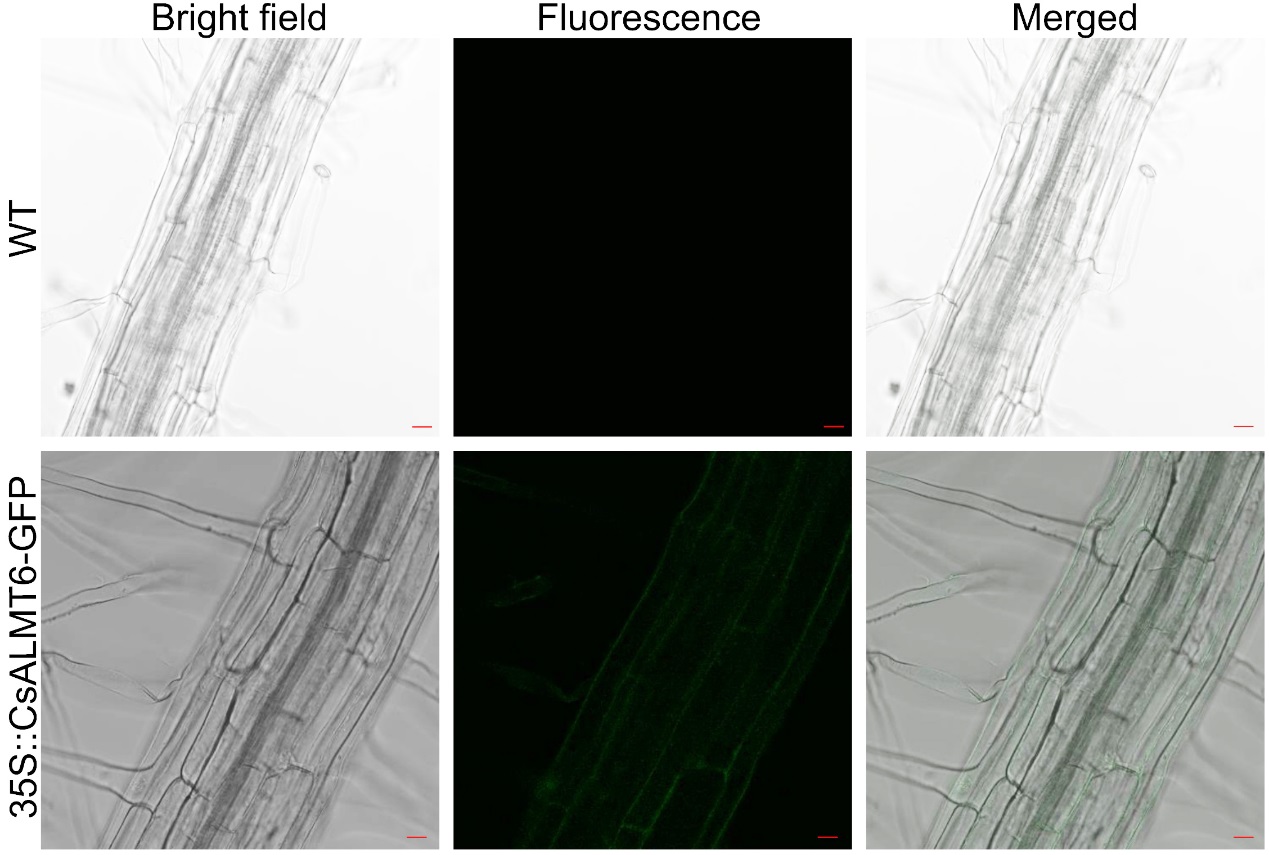


**Fig. S5**


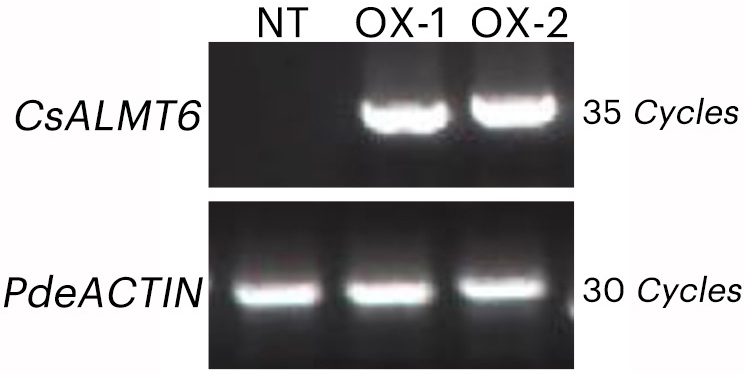


**Fig. S6**


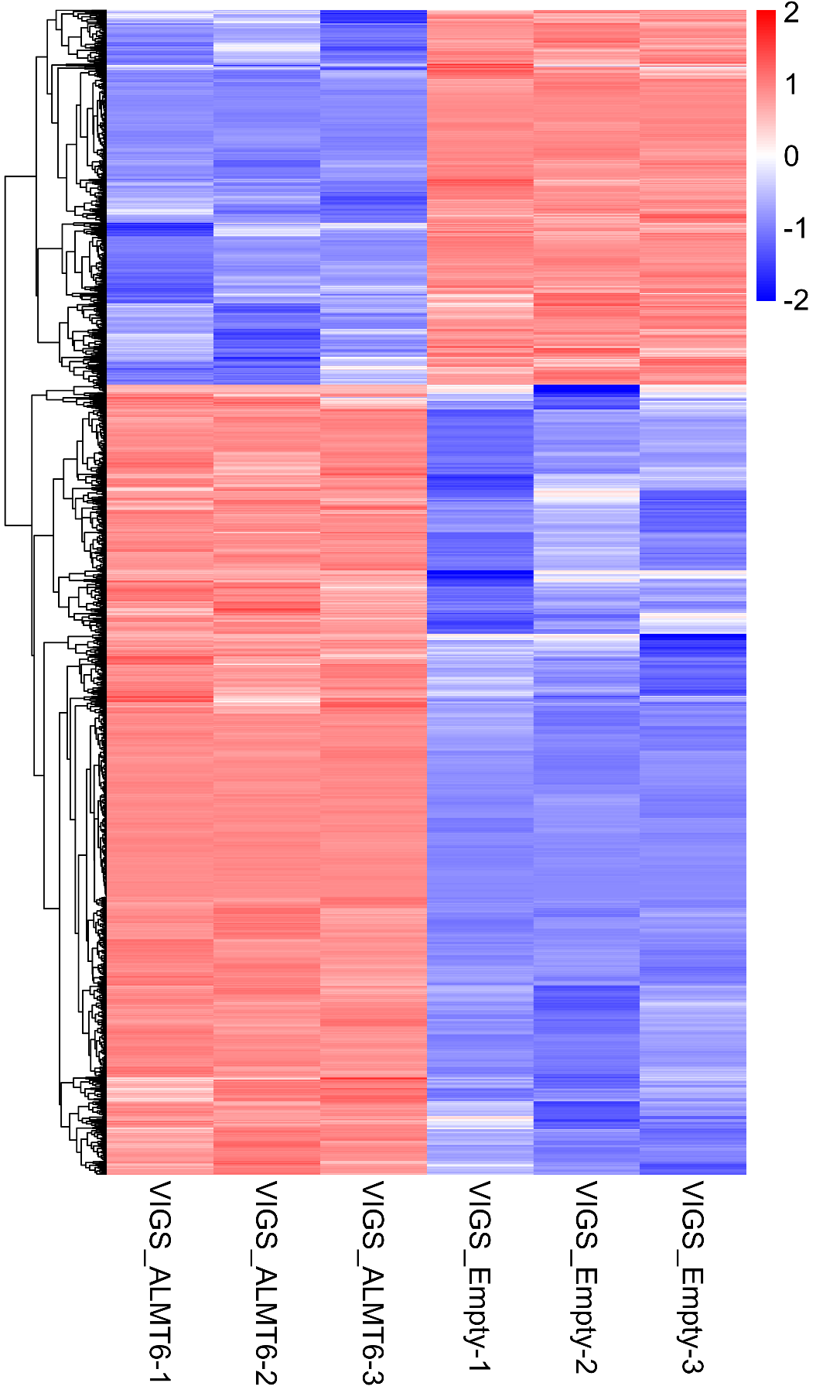


**Fig. S7**

Supplement: Web_Material_uhae353 [file web_material_uhae353.zip › Supplementary Figures.docx]
